# Sexual Dysfunction in Heroin Dependents: A Comparison between Methadone and Buprenorphine Maintenance Treatment

**DOI:** 10.1371/journal.pone.0147852

**Published:** 2016-01-28

**Authors:** Anne Yee, Mahmoud Danaee, Huai Seng Loh, Ahmad Hatim Sulaiman, Chong Guan Ng

**Affiliations:** 1 University Malaya Center of Addiction Sciences, Department of Psychological Medicine, Faculty of Medicine, University of Malaya, Kuala Lumpur, Malaysia; 2 Clinical Academic Unit (Family Medicine), Newcastle University Medicine Malaysia, Nusajaya, Johor, Malaysia; The University of Texas at Austin, UNITED STATES

## Abstract

**Introduction:**

Methadone has long been regarded as an effective treatment for opioid dependence. However, many patients discontinue maintenance therapy because of its side effects, with one of the most common being sexual dysfunction. Buprenorphine is a proven alternative to methadone. This study aimed to investigate sexual dysfunction in opioid-dependent men on buprenorphine maintenance treatment (BMT) and methadone maintenance treatment (MMT). The secondary aim was to investigate the correlation between sexual dysfunction and the quality of life in these patients.

**Methods:**

Two hundred thirty-eight men participated in this cross-sectional study. Four questionnaires were used, the Mini International Neuropsychiatric Interview, Opiate Treatment Index, Malay version of the International Index of Erectile Function 15 (Mal-IIEF-15), and World Health Organization Quality of Life-BREF Scale. Multivariate analysis of covariance was used to examine the relationship between MMT and BMT and the Mal-IIEF 15 scores while controlling for all the possible confounders.

**Results:**

The study population consisted of 171 patients (71.8%) on MMT and 67 (28.2%) on BMT. Patients in the MMT group who had a sexual partner scored significantly lower in the sexual desire domain (p < 0.012) and overall satisfaction (p = 0.043) domain compared with their counterparts in the BMT group. Similarly, patients in the MMT group without a sexual partner scored significantly lower in the orgasmic function domain (p = 0.008) compared with those in the BMT group without a partner. Intercourse satisfaction (p = 0.026) and overall satisfaction (p = 0.039) were significantly associated with the social relationships domain after adjusting for significantly correlated sociodemographic variables.

**Conclusions:**

Sexual functioning is critical for improving the quality of life in patients in an opioid rehabilitation program. Our study showed that buprenorphine causes less sexual dysfunction than methadone. Thus, clinicians may consider the former when treating heroin dependents who have concerns about sexual function.

## Introduction

In 2005, the Malaysian government began the methadone maintenance treatment (MMT) program as a national program, with 10,000 opioid-dependent individuals voluntarily enrolled [1]. Local data have shown that the MMT program has reduced crime rates and high-risk behavior [2] and improved the quality of life [3], social functioning, and employment status [4] of heroin dependents. Although the Malaysian government has strongly supported harm reduction policies, increasing the number of MMT programs remains an immense challenge. This may be due to the methadone’s side effects, which may cause some patients to leave the MMT program. One of the most common side effects of methadone is sexual dysfunction [5].

Numerous studies have been conducted on MMT, and many have found that sexual dysfunction, including hypoactive sexual desire disorder, erectile dysfunction, and orgasmic dysfunction, is common in heroin users and individuals being treated for heroin addiction [5–16]. In a recent meta-analysis, the meta-analytical pooled prevalence for sexual dysfunction among methadone users was 52% (95% confidence interval, 0.39–0.65). Hypoactive sexual desire disorder and low libido were the most prevalent sexual dysfunctions, accounting for 51% of cases [17]. Although reduction of the dose or discontinuation of methadone would be expected to improve sexual function, conflicting results have been obtained in previous studies [5,7]. Other possible confounders, such as depression, older age, psychological disorders, medical conditions such as obesity and viral hepatitis, and unhealthy lifestyle [9,18–20], can have negative effects on sexual function.

Methadone is a slow- and long-acting opiate agonist that stimulates μ-opioid receptors in various areas of the brain. Several hypotheses have been suggested to explain the correlation between methadone use and sexual dysfunction. One well-known hypothesis is that methadone exerts neuroendocrinological effects on the tubero-infundibular and hypothalamic-pituitary-gonadal axes. The chronic stimulation of the μ-opioid receptors by methadone alters the function of the tubero-infundibular axis and the dopaminergic control of prolactin, with a consequential impact on sexual functioning [21]. A high level of circulating prolactin causes the inhibition of the gonadotropin-releasing hormone, which lowers the levels of sex hormones, especially testosterone. Men with low testosterone levels may exhibit a decrease in sexual interest [22]. Furthermore, studies have also shown that methadone inhibits copulatory behavior when administered acutely or chronically in animals, and it decreases socio-sexual interaction without interfering with locomotion [23,24].

Buprenorphine, an alternative to methadone, has recently been increasingly recognized as an effective anti-opioid addiction agent. It is a mixed agonist-antagonist opioid, with a low intrinsic activity and a high affinity for the µ-opioid receptor and no intrinsic activity but a high affinity for the κ-opioid receptor. Histological studies in buprenorphine-administered rats showed no significant changes in the basement membrane, seminiferous tubules, Sertoli cells, interstitial tissue, or sperm compared to a group that received methadone [25]. In humans, however, the results have been contradictory. Some studies have found that, unlike MMT, buprenorphine maintenance treatment (BMT) was not associated with sexual dysfunction. In contrast, other studies have found that erectile dysfunction was equally frequent in the two groups [5,6,16,26]. All these trials, however, had small samples sizes; therefore, the role of buprenorphine in sexual dysfunction in heroin-dependent patients is yet to be determined [5,6].

A recent qualitative study [27] has found that some MMT subjects who experienced sexual dysfunction chose to withdraw from interactions with their partners, which led to conflicts. Such conflicts negatively impacted the rehabilitation. Furthermore, inappropriate reactions to the sexual problems included premature treatment discontinuation under pressure from partners, methadone dose reduction, and use of other illicit drugs to enhance sexual performance [27]. Although sexual dysfunction is not life-threatening, it may often result in withdrawal from sexual intimacy, thereby reducing quality of life [7].

Therefore, we conducted this study to investigate sexual dysfunction in opiate-dependent men on MMT and BMT. We also investigated the correlation and association between sexual dysfunction and quality of life in this group of patients.

## Methods

This cross-sectional study included 238 men who received the MMT or BMT at the University of Malaya Medical Center and University of Malaya Center of Addiction Sciences from September 2013 to September 2014. All patients who met the inclusion criteria were asked to participate. Inclusion criteria were as follows: (a) age ≥18 years, (b) presence of sexual activity (i.e., intercourse, caressing one’s partner without having intercourse, foreplay, and masturbation) within the past 4 weeks, and (c) ability to understand and communicate in English or Bahasa Malaysia to complete the study measurements. Exclusion criteria were as follows: (a) current treatment with antiviral medication for viral hepatitis or HIV, androgen replacement therapy, or phosphodiesterase type 5 inhibitors, (b) use of psychotropic medications other than methadone or buprenorphine, and (c) participation in MMT or BMT for <8 weeks. Written informed consent was obtained from all study participants. Participation in the study was voluntary and confidential, and no remuneration was provided. Both medications were administered and prescribed to patients in the oral tablet form. The present study was conducted in accordance with the Declaration of Helsinki, and the Medical Ethics Committee of University Malaya Medical Center approved (902.17) and monitored the study.

All eligible participants agreed to complete an interview, which included questions on sociodemographic factors such as duration of treatment and dosage of methadone or buprenorphine. The participants were assessed by using the Mini International Neuropsychiatric Interview (M.I.N.I.) and Opiate Treatment Index (OTI). The participants also completed the Malay version of the International Index of Erectile Function 15 (Mal-IIEF-15) and World Health Organization Quality of Life-BREF Scale (WHOQOL-BREF) questionnaires.

### Instruments

#### 1. Mal-IIEF-15

The Mal-IIEF-15 is an easy-to-use, multi-dimensional self-reporting instrument, which is available in multiple languages. It consists of 15 items that evaluate five distinct domains of male sexual function over the preceding 4 weeks [28]. These domains and the corresponding International Index of Erectile Function (IIEF) items explore erectile function (questions 1, 2, 3, 4, 5, and 15), orgasmic function (questions 9 and 10), sexual desire (questions 11 and 12), intercourse satisfaction (questions 6, 7, and 8), and overall satisfaction (questions 13 and 14). Each item is rated on a Likert scale ranging from 0 to 5, with a higher score corresponding to better sexual function. The Malay version of the IIEF has been validated as a reliable instrument for measuring sexual dysfunction in the Malaysian male population. Its Cronbach’s α value is at least 0.74, and the intra-class correlation coefficient is 0.59 [29]. In the present study, a sexual partner was defined according to the IIEF-15 as a subject with whom one has had sexual activity in the past 4 weeks.

#### 2. M.I.N.I

The M.I.N.I. is a face-to-face, short, structured diagnostic interview designed to identify 10 psychiatric disorders: mood disorders (major depression, bipolar disorder, dysthymia, and suicidality), panic disorder, social anxiety disorder, generalized anxiety disorders, obsessive-compulsive disorder, post-traumatic stress disorder, psychotic disorders, substance dependence and abuse, eating disorders, and antisocial personality [30]. The M.I.N.I. has good validity and reliability, and it can be administered in 15–20 minutes [31]. In this study, this instrument was used to rule out any comorbid psychiatric disorders in the patients on methadone maintenance.

#### 3. OTI

The OTI is a multidimensional, structured, face-to-face interview used to evaluate opiate treatment. The OTI consists of objective questions that assess six independent outcome domains: drug use, HIV risk-taking behavior, social functioning, criminality, health status, and psychological adjustment. All questions explore behavior in the month prior to the day of the interview, except for the social functioning section, which covers the preceding 6 months. The drug use domain is examined by collecting information on the last 3 days of drug use for each drug category. For each drug class, the patient is asked to list their three most recent episodes of drug use. A Q score is then calculated by adding the numbers of use episodes reported by the subject and dividing the result by the total of two intervals between the reported use episodes. A higher Q score corresponds to heavier drug use. In the other domains, all questions are scored on a Likert scale ranging from 0 (best) to 5 (worst). The score for each domain is determined by adding the scores for each question. A higher score denotes a greater degree of dysfunction. The OTI typically takes 30 minutes to administer and is one of the evaluation tools used by the MMT program [32].

#### 4. WHOQOL-BREF

The WHOQOL-BREF is an abbreviated version of the World Health Organization Quality of Life-100 assessment. The questionnaire is used to assess an individual's subjective perception of quality of life over the preceding 2 weeks. It consists of 26 questions and primarily covers four domains (physical, psychological, social relationship, and environment). The total score of the WHOQOL-BREF ranges from 0 to 100, with higher scores reflecting higher quality of life. The Malay version of the WHOQOL-BREF has been validated and found to have sound psychometric properties (e.g., good discriminant and construct validities), high internal consistency (0.64–0.80), and good test-retest reliability (0.49–0.88) [33]. This scale was used to measure the quality of life in this group of patients.

### Statistical Analysis

All analyses were conducted with the Statistical Package of Social Sciences, version 22.0 (SPSS, Chicago, IL, USA). Descriptive statistics, including the mean, standard deviation, and frequency, were computed for the baseline characteristics of the patients on MMT and BMT. Demographic characteristics such as age, ethnicity, education level, employment status, marital status, current methadone dose, history of hepatitis, and psychiatric comorbidities were compared between the patients on methadone and buprenorphine. The groups were compared by using the chi-square and Fisher exact tests for categorical variables, independent sample t-test for normally distributed continuous variables, and Mann-Whitney U test for not normally distributed variables. Prior to analyzing continuous variables, normality was tested using the Shapiro-Wilk test. Analysis of covariance was performed using a general linear model (for normally distributed variables) and a generalized linear model (for not normally distributed variables) to compare sexual function in patients on MMT and BMT using ethnicity, hepatitis C status, education level, Q scores for tobacco and amphetamines, and social functioning determined by the OTI as covariates. A pairwise comparison was applied using a method based on the Bonferroni test. We conducted a sub-analysis to further investigate those with and without a sexual partner. Correlations between the Mal-IIEF-15 and WHOQOL-BREF domains were evaluated for those with and without partners using partial correlation analysis with ethnicity, hepatitis C status, education level, Q scores for tobacco and amphetamines, and social functioning determined according to the OTI as covariates. The Fisher r-to-z transformation [34] was also used to assess the significance of the difference between the WHOQOL-BREF and Mal-IIEF-15 correlation coefficients in patients with partners on MMT and BMT. Hierarchical multiple regression was then performed for the entire study population with partners using the WHOQOL-BREF as the dependent variable and the Mal-IIEF-15 as the independent variable. Prior to the regression analysis, the relationship between sociodemographic variables and dependent variables was analyzed, and significantly correlated sociodemographic variables were considered confounding variables in the analysis. All the significant confounding variables, excluding the sexual dysfunction components, were entered into the first block. The independent variables were entered into the second block. Statistical significance was set at p < 0.05 as determined using two-sided tests.

## Results

In this study, 71.8% (n = 171) of the patients were on MMT, and the remaining 28.2% (n = 67) were on BMT. The patients’ demographic and treatment details are shown in Table 1. Patients on MMT and BMT differed in terms of ethnicity (p = 0.02); education level (p < 0.01); hepatitis C status (p < 0.01); and OTI Q scores for tobacco (p = 0.004), amphetamines (p = 0.037), and social functioning (p < 0.01).

**Table 1:** Demographic and Treatment Characteristics of All the Participants.

	MMT (n = 171)	BMT (n = 67)	df	χ^2^, Z,t	p value
**Age**, years, mean ± SD	43.01 ± 9.52	43.43 ± 11.76	101.6	t = -0.261	0.79
**Daily dose**, mg, mean ± SD	77.08 ± 30.94	4.06 ± 2.78	—	—	N/A
**BMI,** kg/m^2^, mean ± SD	23.21 ± 4.85	22.33 ± 5.07	233	t = 1.226	0.23
**Duration of MMT or BMT,** months, mean ± SD	41.48 ± 28.60	50.03 ± 34.99	102.1	Z = -1.78	0.07
**Current partner,** n (%)	114 (66.7)	40 (59.7)	1	χ^2^ = 1.023	0.36
**Marital Status,** n (%)					
** Single**	55 (32.2)	28 (41.8)	3	χ^2^ = 3.344	0.34
** Married**	100 (58.5)	36 (53.7)			
** Divorced**	13 (7.6)	3 (4.5)			
**Ethnic Group,** n (%)					
** Malay**	144 (84.2)	46 (68.7)	3	χ^2^ = 9.475	0.02*
** Chinese**	15 (8.8)	14 (20.9)			
** Indian**	10 (5.8)	7 (10.4)			
** Others**	2 (1.2)	0			
**Religion,** n (%)					
** Islam**	144 (84)	48 (71.6)	4	χ^2^ = 6.284	0.18
** Christianity**	10 (5.8)	7 (10.4)			
** Buddhism**	8 (4.7)	8 (11.9)			
** Hindu**	6 (3.5)	3 (4.5)			
** Others**	3 (1.8)	1 (1.5)			
**Education Level,** n (%)					
** No education**	2 (1.2)	0	3	χ^2^ = 17.386	<0.01**
** Primary**	11 (6.4)	17 (25.4)			
** Secondary**	151 (88.3)	47 (70)			
** Tertiary**	7 (4.1)	3 (4.5)			
**Employed,** n (%)	143 (83.6)	60 (89.6)	1	χ^2^ = 1.348	0.24
**Family history of drug use,** n (%)	41 (24)	15 (22.4)	1	χ^2^ = 0.068	0.79
**HBV+,** n (%)	5 (2.9)	3 (4.5)	1	χ^2^ = 0.358	0.69
**HCV+,** n (%)	58 (33.9)	7 (10.4)	1	χ^2^ = 13.358	<0.01**
**OTI Q scores for drug use domain**^Ϯ^**,** mean ± SD					
** Tobacco**	8.43 ± 6.28	6.50 ± 8.10	-	Z = -2.867	0.004**
** Alcohol**	0.02 ± 0.12	0.14 ± 0.89	-	Z = -0.731	0.465
** Benzodiazepine**	0.01 ± 0.07	0.07 ± 0.52	-	Z = -1.605	0.108
** Marijuana**	0.03 ± 0.33	0.07 ± 0.56	-	Z = -0.172	0.864
** Amphetamines**	0.003 ± 0.024	0.22 ± 1.71	-	Z = -2.081	0.037*
** Heroin**	0.07 ± 0.43	0.13 ± 0.65	-	Z = -0.06	0.947
**Q scores for HIV risk-taking domain,** mean ± SD	3.74 ± 3.47	3.05 ± 3.50	-	Z = -1.511	0.13
**Q scores for criminality domain,** mean ± SD	0.04 ± 0.459	0	-	Z = -0.626	0.531
**Q scores for social functioning domain,** mean ± SD	8.88 ± 5.08	6.72 ± 4.84	-	Z = -3.127	0.002**
**Q scores for health domain,** mean ± SD	0.39 ± 1.13	0.40 ± 0.88		Z = -1.285	0.199
**M.I.N.I. psychiatric disorders**	48 (28.1)	19 (28.4)	5	χ^2^ = 8.713	0.711
** MDD**	16 (9.4)	7 (10.4)			
** ASD**	29 (17)	11 (16.4)			
** Panic disorder**	1 (0.6)	1 (1.5)			
** Dysthymia**	1 (0.6)	0 (0)			
** Bipolar**	1 (0.6)	0 (0)			

ASD, antisocial disorder; BMI, body mass index; BMT, buprenorphine maintenance treatment; df, degrees of freedom; HBV, hepatitis B; HCV, hepatitis C; Mal-IIEF-15, Malay version of the International Index of Erectile Function 15; HIV, human immunodeficiency virus; MDD, major depressive disorder; M.I.N.I., Mini International Neuropsychiatric Interview; MMT, methadone maintenance treatment; OTI, Opioid Treatment Index; SD, standard deviation; t, t-test; χ^2^, chi-square test; Z, z-test.

* p < 0.05

** p < 0.01

^Ϯ^ Based on the Mann-Whitney test

Multivariate analysis of covariance was used to compare the differences in the Mal-IIEF-15 scores between the MMT and BMT groups while controlling for ethnicity, education level, hepatitis C status, Q scores for tobacco and amphetamines, and social functioning OTI domains with pairwise comparisons using Bonferroni multiple testing corrections. Patients in the MMT group, both with and without sexual partners, had lower Mal-IIEF-15 scores than patients in the BMT group. Patients in the MMT group with sexual partners scored significantly lower in the sexual desire (p < 0.012) and overall satisfaction (p = 0.043) domains than patients in the BMT group (Table 2). Similarly, patients in the MMT group without a partner scored significantly lower in the orgasmic function domain (p = 0.008) than patients without a partner in the BMT group (Table 3). An analysis of marital status within the subgroup with sexual partners did not reveal any differences (data not shown).

**Table 2:** Comparison of the Mean Mal-IIEF-15 Domain Scores in Patients with Sexual Partners in the MMT and BMT Groups.

Mal-IIEF-15 domain	MMT (n = 114) mean ± SD	BMT (n = 40) mean ± SD	Mean difference^a^	p value^b^
**Erectile function**	21.77 ± 7.02	21.93 ± 8.48	-0.942	0.506
**Orgasmic function**^**ƚ**^	7.37 ± 2.86	7.40 ± 3.58	-0.07	0.902
**Sexual desire**^**ƚ**^	6.31± 1.57	7.10 ± 1.58	-0.76	<0.012*
**Intercourse satisfaction**	8.81 ± 3.35	9.73 ± 4.38	-1.180	0.086
**Overall satisfaction**^**ƚ**^	7.70 ± 1.92	8.35 ± 1.89	-0.73	0.043*

BMT, buprenorphine maintenance treatment; MMT, methadone maintenance treatment; Mal-IIEF-15, Malay version of the International Index of Erectile Function 15; SD, standard deviation.

* p < 0.05

^a^ Adjusted mean difference with ethnic group, education level, hepatitis C status, social functioning total score, and Q score for tobacco and amphetamines as covariates.

^b^ Adjusted for multiple comparisons using the Bonferroni correction.

^ƚ^ A generalized linear model approach was used because the variables were not normally distributed.

**Table 3:** Comparison of the Mean Mal-IIEF-15 Domain Scores of All Patients without a Sexual Partner in the MMT and BTM Groups.

Mal-IIEF-15 domain	MMT (n = 57) mean ± SD	BMT (n = 27) mean ± SD	Mean difference^a^	p value^b^
**Erectile function**	8.37 ± 7.09	9.37 ± 8.29	-2.587	0.225
**Orgasmic function**^**ƚ**^	2.33 ± 3.23	3.85 ± 3.91	-2.47	0.008**
**Sexual desire**	5.91 ± 2.37	5.26 ± 1.91	0.51	0.373
**Intercourse satisfaction**^**ƚ**^	1.44 ± 3.29	1.96 ± 3.94	-1.118	0.257
**Overall satisfaction**	4.39 ± 2.27	4.48 ± 2.47	-0.12	0.853

BMT, buprenorphine maintenance treatment; MMT, methadone maintenance treatment; Mal-IIEF-15, Malay version of the International Index of Erectile Function 15; SD, standard deviation.

** p < 0.01

^a^ Adjusted mean difference for the covariates (i.e., ethnic group, education level, hepatitis C status, social functioning total score, and Q score for tobacco and amphetamines).

^b^ Adjusted for multiple comparisons using the Bonferroni correction.

^ƚ^ A generalized linear model approach was used because the variables were not normally distributed.

Table 4 shows the partial correlation coefficients between sexual function (Mal-IIEF-15) and the WHOQOL-BREF scores in patients with sexual partners for MMT and BMT separately. In the BMT group, intercourse function and erectile function were positively correlated for all the WHOQOL-BREF domains. Sexual desire correlated significantly with only the psychological (r = 0.444, p < 0.01) and social relationships (r = 0.500, p < 0.01) domains. In the MMT group, overall satisfaction correlated strongly with the psychological (r = 0.309, p < 0.01) and social relationships (r = 0.262, p < 0.01) domains in the WHOQOL-BREF.

**Table 4:** Relationship between Sexual Dysfunction and Quality of Life for All Patients with a Sexual Partner in the MMT and BMT Groups ^a^.

	WHOQOL domain							
	Physical health		Psychological Health		Social relationships		Environment	
**Mal-IIEF-15 domain**	**MMT**	**BMT**	**MMT**	**BMT**	**MMT**	**BMT**	**MMT**	**BMT**
**Erectile function**	0.175	0.363*	0.222*	0.367*	0.162	0.367*	0.186	0.351*
**Orgasmic function**	0.161	0.268	0.211*	0.305	0.195*	0.278	0.215*	0.324
**Sexual desire**	0.075	0.086	0.111	0.444**	0.19	0.5**	0.112	0.283
**Intercourse satisfaction**	0.141	0.433**	0.277**	0.531**	0.206*	0.590**	0.207*	0.455**
**Overall satisfaction**	0.166	0.274	0.309**	0.241	0.262**	0.292	0.186	0.151

BMT, buprenorphine maintenance treatment; MMT, methadone maintenance treatment; Mal-IIEF-15, Malay version of the International Index of Erectile Function 15; WHOQOL, World Health Organization Quality of Life.

* p < 0.05

**p < 0.01

^a^ Adjusted for ethnic group, education level, hepatitis C status, social functioning total score, and Q scores for tobacco and amphetamines using the partial correlation method.

The Fisher r-to-z transformation showed a relationship between psychological and sexual desire (r_MMT_ = 0.111, r_BMT_ = 0.444, Z = 1.93, p = 0.026), and social relationships and sexual desire (r_MMT_ = 0.19, r_BMT_ = 0.5, Z = 1.88, p = 0.03) were significantly different in the MMT and BMT groups. Similarly, the relationships between intercourse and physical health (r_MMT_ = 0.141, r_BMT_ = 0.433, Z = 1.69, p = 0.04), and intercourse and social relationships (r_MMT_ = 0.206, r_BMT_ = 0.590, Z = 2.17, p = 0.015) were also significantly different between the MMT and BMT groups.

Table 5 shows that the overall satisfaction domain was significantly associated with the psychology domain (p = 0.047) in the WHOQOL-BREF, whereas the intercourse satisfaction (p = 0.026) and overall satisfaction (p = 0.039) domains were significantly associated with the social relationships domain after adjusting for significantly correlated sociodemographic variables.

**Table 5:** Linear Regression Analysis of Sexual Dysfunction and Quality of Life for All Patients with a Sexual Partner in the MMT and BMT Groups.

	Physical Health		Psychological Health		Social relationships		Environment	
Variable,n (%)	B (β)	p value	B (β)	p value	B (β)	p value	B (β)	p value
**Erectile Function**	0.017 (0.058)	0.71	-0.031 (-0.1)	0.531	-0.087 (-0.226)	0.139	-0.031 (-0.098)	0.55
**Orgasmic Function**	0.047 (0.067)	0.533	0.01 (0.013)	0.908	0.02 (0.021)	0.84	0.076 (0.098)	0.381
**Sexual Desire**	-0.028 (-0.021)	0.813	0 (0)	0.997	0.257 (0.145)	0.099	0.05 (0.034)	0.721
**Intercourse Satisfaction**	0.033 (0.057)	0.723	0.185 (0.295)	0.074	0.272 (0.351)	0.026*	0.147 (0.226)	0.177
**Overall Satisfaction**	0.154 (0.14)	0.115	0.214 (0.18)	0.047*	0.264 (0.18)	0.039*	0.098 (0.08)	0.386
**Adjusted R**^**2**^	15.9%		11.1%		20.8%		8.2%	

BMT, buprenorphine maintenance treatment; MMT, methadone maintenance treatment

* p < 0.05

## Discussion

In our study, patients with sexual partners in the MMT group had lower scores for sexual desire (p = 0.01) and overall satisfaction (p = 0.043) than patients with sexual partners in the BMT group. Patients in the MMT group without a sexual partner also scored lower in orgasmic function than their counterparts in the BMT group (p = 0.01). In addition, we found that the sexual desire and intercourse domains positively correlated with the WHOQOL-BREF domains, especially the social relationships domain, and these correlations were statistically significant in the BMT group compared with the MMT group.

Patients with sexual partners in the BMT group had higher sexual desire than their counterparts in the MMT group after controlling for all possible confounders. This may indicate that the loss of sexual desire caused by long-term methadone maintenance may be different from that caused by long-term heroin abuse. Sexual desire is defined as the urge to engage in sexual activity [35].Sexual activity is a natural reward in animals, including humans, and the expression of sexual behaviors is based on the influence of sexual excitatory and inhibitory mechanisms in the brain [36]. Although the excitatory mechanisms involve multiple neurotransmitters, dopamine is the one that has been discussed for centuries [36]. The first recognized dopamine-mediated enhancement of sexual behavior in humans occurred when it was noticed that the administration of L-dopa (3,4-dihydroxy-l-phenylalanine), the precursor of dopamine, to men suffering from Parkinson's disease resulted in increased libido and sexual potency [37]. In contrast, endogenous opioid, endocannabinoid, and serotonin systems blunt this excitatory mechanism and act as the sexual inhibitor to induce a restorative state of sexual satiety that presents as a “refractory phase.” This endogenous inhibitory mechanism can also be activated by situational variables such as stress or by drugs such as methadone, which is a slow- and long-acting opioid agonist that works by stimulating μ-opioid receptors [37]. Although normal sexual inhibition keeps individuals from engaging in risky or inappropriate sexual behaviors, excessive central inhibition increases the risk of sexual dysfunction, including inhibited arousal and desire and/or a diminished capacity to achieve sexual gratification [36]. Furthermore, there is some evidence that methadone interferes with the normal production of hypothalamic and pituitary regulatory hormones that increase the serum prolactin level and reduce the gonadotropin releasing hormone level, which indirectly suppresses testosterone production [26]. Testosterone deficiency is accompanied by fatigue, weakness, mood disturbances, and decrease in libido and sexual function [38]. Therefore, the methadone’s effects on sexual behavior are similar to those of anti-androgens [39]. Conversely, buprenorphine, which is a partial opioid agonist of the μ receptor as well as the κ opioid receptor antagonist, induces dopamine release and does not interfere with the sex hormones to the same extent as methadone, causing less reduction of sexual desire [40]. However, sexual desire is not solely dependent on the biological component, and its psychological component is influenced by the interpersonal state (presence or absence of sexual partner) and social context [41]. Accordingly, we only observed changes in sexual desire in the patients who had sexual partners.

An orgasm is defined as a sudden discharge of accumulated sexual excitement during the sexual response cycle causing rhythmic muscular contractions in the pelvic region that are characterized by sexual pleasure. Medical professionals use physiological changes in the body to define an orgasm, whereas psychologists and mental health professionals utilize emotional and cognitive changes [42]. An orgasmic problem is a stressful condition that can prevent people from having sexual and social relationships. In a study by Chekuri et al., nearly half of patients without a sexual partner who were on MMT and suffered from an orgasmic problem stated that they would be in a sexual relationship if they had no orgasmic problem [8]. This may be the reason why the patients remained single and had no sexual partner. We also found that men without a sexual partner who were on MMT had more orgasmic problems than men without a sexual partner on BMT. This result is consistent with those of previous studies [43]. A recent study showed that problems with orgasm actually worsened after 6 months on MMT [44]. The authors argued that this could be due to a variety of psychological and interpersonal factors not monitored during MMT, and the causes remain unknown. Therefore, more research should concentrate on orgasmic problems in this group of patients.

The second objective of our study was to investigate the correlation of sexual dysfunction and quality of life in patients on MMT and BMT. We found that patients on BMT with a sexual partner who had better sexual desire and intercourse scores on the IIEF15 reported a better quality of life, especially in their social relationships. This agrees with the results of an Italian longitudinal survey, in which 3105 (81.5%) MMT and 707 (18.5%) BMT subjects were assessed for social life status based on percentage of subjects who were married or cohabiting. The study found that the social life status was significantly better in the patients receiving BMT (63% and 39% of the BMT and MMT subjects, respectively, were married/cohabiting). The authors concluded that improved psychosocial functioning in the BMT patients could contribute to better reintegration into the community and to enhanced social activity [45]. In contrast, sexual dysfunction caused by MMT interferes with intimate relationships, which may reduce compliance with the therapy and, consequently, its benefits. Patients who experience sexual dysfunction may not have desire or confidence to maintain an intimate affective relationship with their spouse or sexual partner, which in turn can cause tensions in the relationship. Such an attitude may also result in their partners questioning the strength of the relationship, which may ultimately lead to separation [27]. This was also observed in our study, as sexual satisfaction was strongly associated with social relationships even after adjusting for all confounders. Therefore, the problem of diminished sexual function in patients on MMT should be properly addressed.

Several limitations of the present study need to be emphasized. First, presence of response bias cannot be excluded because the topic of sex is considered private among more conservative Malaysian patients, who may have concealed their true feelings because they felt too uncomfortable to reveal them to the researchers. Second, 238 participants constitute a relatively small sample. Third, this cross-sectional study included men who received tertiary care in a hospital-based opioid treatment program. Hence, the results cannot be generalized to all opioid users in the community. Fourth, sexual hormonal assays such as assays for testosterone, free testosterone, prolactin, luteinizing hormone, and follicle-stimulating hormone were not performed in this study owing to financial restrictions. However, previous studies have already shown that patients on MMT have lower testosterone levels than patients on BMT [16,46], which may explain the higher prevalence of sexual dysfunction in patients on MMT. Fifth, the nature of the study design may have resulted in recall bias, possibly affecting the results. Sixth, we did not consider duration of opiate dependence prior to treatment. Finally, the IIEF-15 is primarily a validated instrument for measuring erectile dysfunction in men rather than orgasmic function or sexual desire.

In summary, sexual functioning is critical for improving the quality of life in patients in an opioid rehabilitation program. Our study indicates that the use of buprenorphine is associated with less sexual dysfunction than that of methadone in opioid-dependent patients. Hence, clinicians may consider the former agent when treating heroin dependents with concerns about sexual function.

## Supporting Information

S1 Text All the relevant questionnaires. (PDF)
