# Supplementary material for: Sexual Dysfunction in Heroin Dependents: A Comparison between Methadone and Buprenorphine Maintenance Treatment
Source: PLoS One. 2016 Jan 28;11(1):e0147852. doi: 10.1371/journal.pone.0147852 (PMC4731474; doi:10.1371/journal.pone.0147852)
Supplement: S1 Text — (PDF) [file pone.0147852.s001.pdf]

Questionnaire:

Age

Daily methadone dose (mg)

Duration of methadone maintenance therapy (months)

Frequency of the positive urine rapid drug test/year

Other current (12 months) substance use:

- Tobacco
- Alcohol
- Benzodiazepine
- Cannabis
- Stimulants
- Heroin

Prolactin level

Testosterone level

Alanine aminotransferase

HIV status

Hep B status

Hep C status

SECTION II : DRUG USE

** Note for all categories, if the subject responds that their last use of drug was more than a month ago, score zero for that category. Do not include use on day of interview.*

These questions are about your use of Heroin, Opiate, Alcohol, Marijuana, Tranquilliser, Hallucinogens and Tobacco.

		Heroin	Other Opiate	Alcohol	Marijuana	Tranquil-lisers	Halluci-nogens or ATS	Tobacco
1	On what day did you last use?							
2	How many did you have on that day?							
3	On which day before that did you use?							
4	How many did you have on that day?							
5	When was the day before that?							
	q1=							
	q2=							
	t1=							
	t2=							
	Q							

SECTION III : INJECTING AND SEXUAL PRACTICES

These question are about the way you use drug and you recent sexual behaviour. Any information you provide is completely confidential. (Please circle one)

1. Injecting

		Hasn't hit up	Once a week or less	More than once a week (but less than once a day)	Once a day	2-3 times a day	More than 3 times a day
1	How many times have you hit up (ie injected any drugs) in the last month?	0	1	2	3	4	5

** If subject hasn't injected in the last month, score zero for the Drug Use section, and go to question 7*

		No times	One time	Two times	3 - 5 times	6 - 10 times	More than 10 times
2	How many times in the last month have you used a needle after someone else had used it?	0	1	2	3	4	5

		None	One person	Two people	3 - 5 people	6 - 10 people	More than 10 people
3	How many different people have used a needle before you in last month?	0	1	2	3	4	5

		No times	One time	Two times	3 - 5 times	6 - 10 times	More than 10 times
4	How many times in the last month has someone used a needle after you have used it?	0	1	2	3	4	5

		Doesn't re-use	Every time	Often	Sometimes	Rarely	Never
5	How often, in the last month, have you cleaned needles before re-using them?	0	1	2	3	4	5
6	Before using needles again, how often in the last month did you use bleach to clean them?	0	1	2	3	4	5

Drug use sub total

2. Sexual behaviour

		None	One person	Two people	3 - 5 people	6 - 10 people	More than 10 people
7	How many people, including clients, have you had sex with in the last month?	0	1	2	3	4	5

** If no sex in the last month, score zero for sexual behavioural section and go to section Social Functioning*

		No regular partner/ no penetrative Sex	Every time	Often	Sometimes	Rarely	Never
8	How often have you used condoms when having sex with your regular partner(s) in the last month?	0	1	2	3	4	5

		No casual partner/ no penetrative sex	Every time	Often	Sometimes	Rarely	Never
9	How often did you used condoms when you had sex with casual partners in the last month?	0	1	2	3	4	5

		No paid sex/ no penetrative sex	Every time	Often	Sometimes	Rarely	Never
10	How often have you used condoms when you have been paid for sex in the last month?	0	1	2	3	4	5

		No time	One time	Two times	3 - 5 times	6 - 10 times	More than 10 times
11	How many times did you have anal sex in the last month?	0	1	2	3	4	5

Sexual Behaviour sub total

Total Score

SECTION IV : SOCIAL FUNCTIONING

These next few questions concern the social aspects of your life (things like jobs, friends, etc)

		**One**	**Two**	**Three**	**Four**	**Five or more**
1	How many different places have you lived in over the last six months?	0	1	2	3	4
		**None of the time**	**Some of the time**	**Half of the time**	**Most of the time**	**All of the time**
2	How much of the last six months have you been employed?	0	1	2	3	4
		**One**	**Two**	**Three**	**Four or More**	**None**
3	How many different full time jobs have you had in the last six months?	0	1	2	3	4
		**Never / N/A**	**Rarely**	**Sometimes**	**Often**	**Very often**
4	How often in the last six months have you had conflict with your relatives?	0	1	2	3	4
5	How often in the last six months have you had conflict with your partner(s)?	0	1	2	3	4
		**Never**	**Rarely**	**Sometimes**	**Often**	**Very often / NA**
6	How often in the last six months have you had conflict with your friends?	0	1	2	3	4
		**Four or more**	**Three**	**Two**	**One**	**None**
7	About how may close friends would you estimate that you have? (INCLUDE PARTNER)	0	1	2	3	4
		**Very satisfied / NA**	**Satisfied**	**Reasonably OK**	**Not satisfied**	**Very unsatisfied**
8	When you are having problems, are you satisfied with the support you get from your friends?	0	1	2	3	4
		**Very often**	**Often**	**Sometimes**	**Rarely**	**Never / NA**
9	About how often do you see your friends?	0	1	2	3	4
		**All of them**	**More than half**	**About a half**	**Less than half**	**None / NA**
10	How many of the people you hang around with now have you known for more than six months?	0	1	2	3	4
		**None of the time**	**Some of the time**	**Half of the time**	**Most of the time**	**All of the time**
11	How much of the last six month have you been living with anyone who uses heroin?	0	1	2	3	4
		**None**	**Less than half**	**About a half**	**More than half**	**All of them**
12	How many of the people you hang around with now are users (include partner)?	0	1	2	3	4

Social functioning total ☐

SECTION V : CRIME

These section is concerned with crimes that you may have committed. Any information that you give is completely confidential.
We are interested in the number of time that you committed a crime, not the number of time you've been caugh.
(Please circle one)

Property Crime

		No property crime	Less than once a week	Once a week	More than once a week (but less than daily)	Daily
1	How often, on average, during last month have you committed a property crime?	0	1	2	3	4

Tick type of crimes committed

Break & enter ☐ Stealing a prescription pad ☐ Shoplifting ☐ Other (specify) ☐

Receiving stolen goods ☐ Stealing a car ☐ Robbery without violence ☐

Dealing

		No drug dealing	Less than once a week	Once a week	More than once a week (but less than daily)	Daily
2	How often, on average, during last month have you sold drug to someone?	0	1	2	3	4

Tick type of drugs dealt

Heroin ☐ Cocaine ☐ Hallucinogens ☐ Tranquillisers ☐

Marijuana ☐ Speed ☐ Barbiturates ☐ Other (specify) ☐

Fraud

		No fraud	Less than once a week	Once a week	More than once a week (but less than daily)	Daily
3	How often, on average, during last month have you committed a fraud?	0	1	2	3	4

Tick type of fraud committed

Forging cheques ☐ Using someone else's card ☐ Other (specify) ☐

Forging prescriptions ☐ Social security scams ☐

Crimes involving violence

		No violent crime	Less than once a week	Once a week	More than once a week (but less than daily)	Daily
4	How often, on average, during last month have you committed a crime involving violence?	0	1	2	3	4

Tick type of drugs dealt

Assault ☐ Armed robbery ☐ Manslaughter ☐ Other (specify) ☐

Using violence in a robbery ☐ Murder ☐ Rape ☐

Current convictions

1 Are you currently facing charges? ☐ Yes ☐ No

Crime total ☐

SECTION VI : HEALTH

These questions are about your health. Please tick any check boxes if you have had any of these problems over the last month

General

1 Fatigue/energy loss	☐	6 Night sweats	☐	11 Teeth problems	☐
2 Poor appetite	☐	7 Swollen glands	☐	12 Eye/vison problems	☐
3 Weight loss/underweight	☐	8 Jaundice	☐	13 Ear/hearing problems	☐
4 Trouble sleeping	☐	9 Bleeding easily	☐	14 Cuts needing stitches	☐
5 Fever	☐	10 Bruising easily	☐		

Sub total ☐

Injection related problems

1 Overdose	☐	3 Wheezing	☐	5 Difficulty injecting	☐
2 Abscesses/infections from injecting	☐	4 Prominent scarring/bruising	☐		

Sub total ☐

Cardio/respiratory

1 Persistent cough	☐	4 Wheezing	☐	7 Chest pains	☐
2 Coughing	☐	5 Sore throat	☐	8 Heart flutters/racing	☐
3 Coughing up blood	☐	6 Shortness of breath	☐	9 Swollen ankles	☐

Sub total ☐

Genito-urinary

1 Painful urination	☐	3 Discharge from penis/vagina	☐
2 Loss of sex urge	☐	4 Rash on/around penis/vagina	☐

Sub total ☐

Gynecological *(Women only) (In the last few month)*

1 Irregular period	☐	2 Miscarriage	☐

Sub total ☐

Musculo-skeletal

1 Joint pains/stiffness	☐	2 Broken bones	☐	3 Muscle pain	☐

Sub total ☐

Neurological

1 Headaches	☐	4 Numbness/tingling	☐	7 Difficulty walking	☐
2 Blackouts	☐	5 Dizziness	☐	8 Head injury	☐
3 Tremors (Shakes)	☐	6 Fits/seizures	☐	9 Forgetting things	☐

Sub total ☐

Gastro intestinal

1 Nausea	☐	4 Stomach pains	☐	7 Diarrhoea	☐
2 Vomiting	☐	5 Constipation	☐		

Sub total ☐

Health total ☐

DRUG SUBSTITUTION THERAPY PROGRAM

WHOQOL-BRIEF

Pusat ______________________

Tarikh penilaian : [ ][ ] – [ ][ ] – [ ][ ]

Nama Doktor: ______________________

Penilaian: 0 bulan / 6 bulan / 12 bulan

Nama Kaunselor: ______________________

(untuk kegunaan pejabat sahaja)

Nama Pesakit : [ ]

No. K.P. Pesakit (Baru) : [ ][ ][ ][ ][ ][ ] – [ ][ ] – [ ][ ][ ][ ] (Lama) [ ] – [ ][ ][ ][ ][ ][ ][ ]

No. I.D Pesakit : [ ][ ][ ][ ][ ][ ][ ][ ]

Sila baca setiap soalan, nyata dan nilai perasaan anda dengan membulatkan nombor di setiap soalan berkenaan mengikut skala yang diberikan

		Sangat tidak berpuas hati	Tidak berpuas hati	Sederhana	Berpuas hati	Sangat berpuas hati
1 (G1)	Sejauh manakah anda berpuas hati dengan kesihatan anda?	1	2	3	4	5

		Sangat tidak baik	Tidak baik	Sederhana	Baik	Sangat Baik
2 (G4)	Bagaimana anda menilai kualiti kehidupan anda?	1	2	3	4	5

Soalan-soalan berikutnya bertanyakan ***setakat mana*** *anda telah menghidap sesuatu perkara* ***dalam 2 minggu yang lepas***

		Tiada langsung	Sedikit sahaja	Sederhana	Sangat banyak	Teramat banyak
3 (F1.4)	Setakat manakah anda berasa kesakitan (fizikal) menghalang anda dari melakukan apa yang anda perlu lakukan?	1	2	3	4	5
4 (F11.3)	Berapa kerapkah rawatan perubatan yang anda perlu untuk berfungsi dalam kehidupan harian anda?	1	2	3	4	5
5 (F4.1)	Setakat manakah anda menikmati keseronokan dalam hidup anda?	1	2	3	4	5
6 (F24.2)	Setakat manakah anda rasa hidup anda bermakna?	1	2	3	4	5

		Tiada langsung	Sedikit sahaja	Sederhana	Sangat	Teramat
7 (F5.3)	Berapa baikkah anda dapat memberi tumpuan?	1	2	3	4	5
8 (F16.1)	Berapa selamatkah anda rasa dalam kehidupan seharian anda?	1	2	3	4	5
9 (F22.1)	Berapa sihatkah persekitaran fizikal anda?	1	2	3	4	5

Soalan-soalan berikutnya bertanyakan ***bagaimana sempurnanya*** *anda mengalami atau berupaya melakukan sesuatu perkara* ***dalam 2 minggu yang lepas***

		Tiada langsung	Sedikit sahaja	Sederhana	Kebanyak-kanya	Sepenuhnya
10 (F2.1)	Setakat mana anda mempunyai cukup tenaga untuk menjalani kehidupan harian anda?	1	2	3	4	5
11 (F7.1)	Sejauh manakah anda dapat menerima rupa paras dan bentuk tubuh badan anda?	1	2	3	4	5
12 (F18.1)	Sejauh manakah anda mempunyai wang yang cukup untuk memenuhi keperluan anda?	1	2	3	4	5
13 (F20.1)	Setakat manakah kemudahan bagi anda untuk mendapatkan maklumat yang diperlukan dalam kehidupan harian?	1	2	3	4	5
14 (F21.1)	Setakat mana anda mempunyai peluang untuk aktiviti riadah?	1	2	3	4	5

Sila baca setiap soalan, nyata dan nilai perasaan anda dengan membulatkan nombor di setiap soalan berkenaan mengikut skala yang diberikan

		Sangat tidak baik	Tidak baik	Sederhana	Baik	Sangat baik
15 (F9.1)	Sebaik manakah keupayaan anda boleh bergerak dari satu tempat ke satu tempat yang lain?	1	2	3	4	5

Soalan-soalan berikut bertanyakan tentang perasaan anda terhadap beberapa aspek tertentu dalam kehidupan anda ***sepanjang 2 minggu yang lepas***

		Sangat tidak berpuas hati	Tidak berpuas hati	Sederhana	Berpuas hati	Sangat berpuas hati
16 (F3.3)	Adakah anda berpuas hati dengan tidur anda?	1	2	3	4	5
17 (F10.3)	Adakah anda berpuas hati dengan keupayaan anda melaksanakan aktiviti kehidupan harian anda?	1	2	3	4	5
18 (F12.4)	Adakah anda berpuas hati dengan keupayaan anda dalam pekerjaan	1	2	3	4	5
19 (F6.3)	Adakah anda berpuas hati dengan diri anda?	1	2	3	4	5
20 (F13.3)	Adakah anda berpuas hati dengan perhubungan peribadi anda?	1	2	3	4	5
21 (F15.3)	Adakah anda berpuas hati dengan perhubungan seks dengan pasangan anda?	1	2	3	4	5
22 (F14.4)	Adakah anda berpuas hati dengan sokongan yang anda dapati dari kawan-kawan anda?	1	2	3	4	5
23 (F17.3)	Adakah anda berpuas hati dengan keadaan tempat tinggal anda?	1	2	3	4	5
24 (F19.3)	Adakah anda berpuas hati dengan kemudahan mendapatkan perkhidmatan kesihatan?	1	2	3	4	5
25 (F23.3)	Adakah anda berpuas hati dengan pengangkutan anda?	1	2	3	4	5

Soalan berikut merujuk kepada kekerapan anda merasa atau mengalami sesuatu emosi ***sepanjang 2 minggu yang lepas***

		Tidak pernah	Jarang-jarang	Kerap	Sangat kerap	Sentiasa
26 (F8.1)	Berapa kerapkah anda mempunyai perasaan-perasaan negatif seperti susah hati, kecewa, kegelisahan atau kemurungan?	1	2	3	4	5

Adakah seseorang telah membantu anda mengisi borang ini? ....

Berapa lamakah masa yang diambil untuk mengisi borang ini? ....

Adakah anda mempunyai sebarang komen mengenai penilaian ini?

....

....

....

....

TERIMA KASIH ATAS KERJASAMA DAN BANTUAN ANDA

M.I.N.I.

Mini International Neuropsychiatric Interview

Versi Indonesia 5.0.0

DSM-IV

Y. Lecrubier, E. Weiller, T. Hergueta, P. Amorim, L.I. Bonora, J.P. Lépine
Hôpital de la Salpétrière - Paris - FRANCE.

D. Sheehan, J. Janavs, R. Baker, K.H. Sheehan, E. Knapp, M. Sheehan
University of South Florida - Tampa - USA.

© 1992, 1994, 1998 Sheehan DV & Lecrubier Y.

Hak cipta dimiliki. Tiada satu bahagian pun dari dokumen ini yang boleh disalin atau diedar dalam apa-apa bentuk saja, atau dengan apa-apa cara pun, elektronik atau mekanik, termasuk membuat salinan, atau melalui apa-apa sistem penyimpanan atau pengambilan maklumat, tanpa keizinan bertulis daripada penulis. Penyelidik dan klinisian yang bekerja dalam lingkungan bukan mencari untung atau milik awam (termasuk universiti, hospital amal dan institusi kerajaan) boleh membuat salinan dari bahan M.I.N.I. bagi penggunaan klinikal dan penyelidikan mereka.

M.I.N.I. 1.0.0 / **Versi Indonesia / DSM-IV / Kini**

NAMA PESAKIT :	______________	*NOMBOR PESAKIT :*	______________
TARIKH LAHIR :	______________	*Waktu Mula Wawancara :*	______________
NAMA PEWAWANCARA :	______________	*Waktu Tamat Wawancara :*	______________
TARIKH WAWANCARA :	______________	*JUMLAH WAKTU :*	______________

MODUL	JANGKA WAKTU	
A. EPISOD TEKANAN UTAMA	Kini (2 minggu yang lalu) + Seumur hidup	
A'. EPISOD TEKANAN UTAMA dengan gambaran melankolia	Kini (2 minggu yang lalu)	Secara pilihan
B. DISTIMIA	Kini (2 tahun yang lalu)	
C. BUNUH DIRI	Kini (sebulan yang lalu)	
D. EPISOD (HIPO)MANIK	Kini + Seumur hidup	
E. GANGGUAN PANIK	Seumur hidup + Kini (sebulan yang lalu)	
F. AGORAFOBIA	Kini	
G. FOBIA SOSIAL	Kini (sebulan yang lalu)	
H. GANGGUAN OBSESI-KOMPULSI	Kini (sebulan yang lalu)	
I. GANGGUAN TEKANAN SELEPAS TRAUMA	Kini (sebulan yang lalu)	Secara pilihan
J. PERGANTUNGAN KEPADA ALKOHOL/PENYALAHGUNAAN ALKOHOL	Kini (12 bulan yang lalu)	
K. PERGANTUNGAN KEPADA BAHAN/PENYALAHGUNAAN BAHAN (bukan alkohol)	Kini (12 bulan yang lalu)	
L. GANGGUAN PSIKOTIK	Seumur hidup + Kini	
M. ANOREXIA NERVOSA	Kini (3 bulan yang lalu)	
N. BULIMIA NERVOSA	Kini (3 bulan yang lalu)	
O. GANGGUAN KEBIMBANGAN MENYELURUH	Kini (3 bulan yang lalu)	
P. GANGGUAN SIKAP ANTISOSIAL	Seumur hidup	Secara pilihan

ARAHAN UMUM

M.I.N.I. direka sebagai wawancara berstruktur singkat untuk gangguan psikiatrik utama Aksis I dalam DSM-IV dan ICD-10. Kesahan dan kebolehpercayaan penyelidikan telah dilakukan dengan membandingkan M.I.N.I. dengan SCID-P dan CIDI. Keputusannya menunjukkan bahawa M.I.N.I. mempunyai skor kesahan dan kebolehpercayaan yang tinggi, tetapi dapat dilaksanakan dalam waktu yang jauh lebih singkat (min 18.7 ± 11.6 minit, median 15 minit) berbanding dengan kedua-dua bahan rujukan tersebut. Ia dapat digunakan oleh klinisian, selepas suatu latihan singkat. Pewawancara biasa memerlukan latihan yang lebih terperinci.

- **Wawancara:**
  Demi memastikan wawancara dapat dilakukan sesingkat mungkin, beritahu pesakit bahawa anda akan menjalankan wawancara klinikal yang lebih berstruktur daripada biasanya, dengan pertanyaan yang sangat spesifik tentang masalah psikologi yang memerlukan jawapan ya atau tidak.

- **Format umum:**
  M.I.N.I. dibahagikan kepada **modul-modul** yang ditandai huruf, masing-masing berkaitan dengan satu kategori diagnostik.
  - Pada permulaan setiap modul (kecuali modul gangguan psikotik), pertanyaan penapisan yang berkaitan dengan kriteria utama gangguan itu diletakkan dalam **kotak kelabu.**
  - Pada akhir setiap modul, **kotak diagnosis** membolehkan klinisian mengisi sama ada kriteria diagnostik dipenuhi.

- **Perjanjian:**
  Ayat-ayat yang ditulis dengan «huruf biasa» harus dibacakan kepada pesakit sebagaimana yang tertera untuk menstandardkan penilaian kriteria diagnostik.
  Ayat-ayat yang ditulis dengan « HURUF BESAR » tidak dibacakan kepada pesakit. Ini adalah arahan kepada pewawancara untuk membantu mendapatkan algoritma diagnostik.
  Ayat-ayat yang ditulis dengan « **huruf tebal** » menunjukkan jangka waktu yang diteliti. Pewawancara membacakan ayat-ayat tersebut sebanyak yang diperlukan. Hanya gejala yang terjadi menjelang jangka waktu yang dinyatakan harus dipertimbang untuk mendapatkan respon.
  Frasa-frasa (dalam kurungan) merupakan contoh klinikal gejala. Frasa-frasa ini dapat dibacakan kepada pesakit untuk menjelaskan pertanyaan.
  Jawapan dengan tanda panah di atasnya (➔) menunjukkan bahawa salah satu kriteria yang diperlukan untuk diagnosis tidak dipenuhi. Dalam hal ini, pewawancara harus pergi ke akhir modul untuk membulatkan « **TIDAK** » pada semua kotak diagnostik dan melanjut ke modul berikutnya.
  Jika istilah-istilah dipisahkan dengan *garis miring (/)*, pewawancara hanya membacakan gejala yang terdapat pada pesakit (misalnya, pertanyaan A3).

- **Arahan penilaian:**
  Semua pertanyaan yang dibacakan harus dinilai. Penilaian dilakukan di sebelah kanan setiap pertanyaan dengan membulatkan YA atau TIDAK.
  Klinisian harus memastikan bahawa setiap dimensi pertanyaan dipertimbangkan oleh pesakit (misalnya: jangka waktu, kekerapan, keparahan, « dan/atau » alternatif).
  Gejala yang diakibatkan oleh penyebab organik atau penggunaan alkohol atau dadah, tidak diberi kod positif dalam M.I.N.I.. M.I.N.I. Plus mempunyai pertanyaan yang menyelidiki isu-isu ini.

Untuk pertanyaan, cadangan, permintaan latihan atau maklumat tentang pembaruan M.I.N.I., sila hubungi:

Yves LECRUBIER, M.D./
Thierry HERGUETA, PsyD
INSERM U302
Hôpital de la Salpétrière
47, boulevard de l'Hôpital
F. 75651 PARIS - FRANCE
tel : +33 (0) 1 42 16 16 59
fax : +33 (0) 1 45 85 28 00
e-mail : hergueta@ext.jussieu.fr

David SHEEHAN, M.D., M.B.A.
University of South Florida
Institute for Research in Psychiatry
3515 East Fletcher Avenue
Tampa , FL USA 33613-4788
tel : +1 813 974 4544
fax : +1 813 974 4575
e-mail : dsheehan@com1.med.usf.edu

→ Bererti : PERGI KE KOTAK(-KOTAK) DIAGNOSTIK DARI MODUL INI, BULATKAN TIDAK DALAM SEMUA KOTAK DAN PINDAH KE MODUL BERIKUTNYA

A. EPISOD TEKANAN UTAMA

A1	Adakah anda secara konsisten berasa tertekan atau sedih, hampir sepanjang hari, hampir setiap hari, bagi dua minggu yang lalu?	TIDAK YA	1
A2	Dalam dua minggu yang lalu, adakah anda kurang berminat terhadap kebanyakan hal atau kurang biasa menikmati hal-hal yang biasanya anda nikmati?	TIDAK YA	2
	ADAKAH A1 ATAU A2 DIBERI KOD YA ?	→ TIDAK YA	
A3	**Dalam dua minggu yang lalu, apabila anda berasa tertekan dan tidak berminat:**		
a	Adakah nafsu makan anda berkurang atau meningkat hampir setiap hari atau adakah berat badan anda meningkat atau menurun tanpa bermaksud demikian? (misalnya, ± 5 % dari berat badan atau ± 3.5 kg untuk seseorang dengan berat 70 kg dalam sebulan)<br>JIKA YA UNTUK SALAH SATU, BERI KOD YA	TIDAK YA	3
b	Adakah anda mengalami kesulitan tidur hampir setiap malam (kesulitan untuk mula tidur, terbangun di tengah malam atau terbangun pada awal pagi, atau tidur berlebihan)?	TIDAK YA	4
c	Adakah anda bercakap atau bergerak lebih perlahan daripada biasanya, atau adakah anda gelisah, tidak tenang atau mengalami kesulitan untuk berduduk diam hampir setiap hari?	TIDAK YA	5
d	Adakah anda berasa penat atau tidak bertenaga, hampir setiap hari?	TIDAK YA	6
e	Adakah anda berasa tidak berharga atau bersalah, hampir setiap hari?	TIDAK YA	7
f	Adakah anda berasa sukar untuk menumpukan perhatian atau mengambil keputusan, hampir setiap hari?	TIDAK YA	8
g	Adakah anda berulang kali berniat untuk menyakiti diri sendiri, ingin bunuh diri atau berharap bahawa anda mati?	TIDAK YA	9
A4	ADAKAH 3 ATAU LEBIH JAWAPAN A3 DIBERI KOD YA?<br>(ATAU 4 JAWAPAN A3 JIKA A1 ATAU A2 DIBERI KOD TIDAK)	TIDAK YA<br>*EPISOD TEKANAN UTAMA KINI*	
	JIKA PESAKIT MEMENUHI KRITERIA UNTUK EPISOD TEKANAN UTAMA KINI:		
A5a	Selama hidup anda, pernahkah anda selama dua minggu atau lebih berasa tertekan atau tidak berminat terhadap kebanyakan hal, dan mengalami masalah yang baru kita bincangkan?	→ TIDAK YA	10
b	Adakah anda tidak mengalami tekanan dan/atau kehilangan minat dalam selang waktu sekurang-kurangnya 2 bulan di antara episod terkini dan episod lalu anda?	TIDAK YA	11
	ADAKAH A5b DIBERI KOD YA?	TIDAK YA<br>*EPISOD TEKANAN UTAMA LALU*	

A'. EPISOD TEKANAN UTAMA DENGAN GAMBARAN MELANKOLIA (secara pilihan)

JIKA PESAKIT DIBERI KOD POSITIF UNTUK EPISOD TEKANAN UTAMA (A4 = YA), PERIKSALAH HAL BERIKUT:

A6 a ADAKAH A2 DIBERI KOD YA ? TIDAK YA 12

b Dalam waktu yang paling parah dalam episod tekanan kini, adakah anda hilang keupayaan untuk berespon terhadap hal-hal yang dulunya memberikan rasa senang, atau menggembirakan anda? TIDAK YA 13
JIKA TIDAK: Jika sesuatu yang baik berlaku, adakah hal itu tidak dapat membuat anda berasa lebih baik, walaupun buat sementara waktu?

ADAKAH A6a ATAU A6b DIBERI KOD YA? → TIDAK YA

Dalam dua minggu yang lalu, apabila anda berasa tertekan dan tidak berminat:

A7 a Adakah anda berasa sedih yang berbeda dari kesedihan apabila seseorang yang rapat dengan anda meninggal? TIDAK YA 14

b Adakah anda hampir setiap hari berasa lebih buruk di sebelah pagi? TIDAK YA 15

c Adakah anda terbangun 2 jam lebih awal dari waktu bangun yang biasa dan menghadapi kesulitan untuk tidur kembali, hampir setiap hari? TIDAK YA 16

e ADAKAH A3c DIBERI KOD YA? TIDAK YA 17

d ADAKAH A3a DIBERI KOD YA (HANYA ANOREXIA ATAU PENURUNAN BERAT BADAN)? TIDAK YA 18

f Adakah anda berasa bersalah yang berlebihan atau tidak seimbang dengan kenyataan yang ada? TIDAK YA 19

ADAKAH 3 ATAU LEBIH JAWAPAN A7 DIBERI KOD YA?

TIDAK	YA
EPISOD TEKANAN UTAMA Dengan Gambaran Melankolia KINI	

→ BERERTI : PERGI KE KOTAK(-KOTAK) DIAGNOSTIK DARI MODUL INI, BULATKAN TIDAK DALAM SEMUA KOTAK DAN PINDAH KE MODUL BERIKUTNYA

B. DISTIMIA

JIKA GEJALA PESAKIT KINI MEMENUHI KRITERIA UNTUK EPISOD TEKANAN UTAMA, JANGAN PERIKSA MODUL INI

B1	Adakah anda berasa sedih, murung atau tertekan hampir sepanjang waktu dalam dua tahun yang lalu?	→ TIDAK	YA	20
B2	Adakah tempoh ini diselingi oleh perasaan yang baik (tidak tertekan) selama dua bulan atau lebih?	TIDAK	→ YA	21
B3	**Apabila anda berasa tertekan hampir sepanjang waktu tersebut:**			
a	Adakah nafsu makan anda berubah secara ketara?	TIDAK	YA	22
b	Adakah anda menghadapi kesulitan untuk tidur atau tidur terlalu banyak?	TIDAK	YA	23
c	Adakah anda berasa penat atau tidak bertenaga?	TIDAK	YA	24
d	Adakah anda kehilangan keyakinan diri?	TIDAK	YA	[illegible]
e	Adakah anda menghadapi kesulitan untuk menumpukan perhatian atau mengambil keputusan?	TIDAK	YA	26
f	Adakah anda berasa tidak berharapan?	TIDAK	YA	27
	ADAKAH 2 ATAU LEBIH JAWAPAN B3 DIBERI KOD YA?	→ TIDAK	YA	
B4	Adakah gejala tekanan itu menyebabkan anda tertekan atau secara ketara mengganggu keupayaan anda untuk bekerja, dalam hubungan sosial, atau dalam hal-hal penting yang lain?	→ TIDAK	YA	28
	ADAKAH B4 DIBERI KOD YA?	TIDAK	YA	

TIDAK	YA
DISTIMIA KINI	

→ BERERTI : PERGI KE KOTAK(-KOTAK) DIAGNOSTIK DARI MODUL INI, BULATKAN TIDAK DALAM SEMUA KOTAK DAN PINDAH KE MODUL BERIKUTNYA

C. BUNUH DIRI

Dalam satu bulan yang lalu, adakah anda:

C1	Berfikir bahawa lebih baik mati atau mengharapkan kematian?	TIDAK	YA	1
C2	Ingin mencederakan diri anda?	TIDAK	YA	2
C3	Berfikir tentang bunuh diri?	TIDAK	YA	3
C4	Mempunyai rancangan untuk bunuh diri?	TIDAK	YA	4
C5	Cuba bunuh diri?	TIDAK	YA	5

Sepanjang hayat anda:

C6	Adakah anda pernah melakukan percubaan bunuh diri?	TIDAK	YA	6

ADAKAH SEKURANG-KURANGNYA 1 DARIPADA YANG DI ATAS DIBERI KOD YA? TIDAK YA

BUNUH DIRI KINI

JIKA YA, TENTUKAN PARAS RISIKO BUNUH DIRI SEPERTI BERIKUT:

C1 atau C2 atau C6 = YA : RENDAH
C3 atau (C2 +C6) = YA : SEDERHANA
C4 atau C5 atau (C3 + C6) = YA : TINGGI

RENDAH ☐
SEDERHANA ☐
TINGGI ☐

→ BERERTI : PERGI KE KOTAK(-KOTAK) DIAGNOSTIK DARI MODUL INI, BULATKAN TIDAK DALAM SEMUA KOTAK DAN PINDAH KE MODUL BERIKUTNYA

D. EPISOD (HIPO)MANIK

D1 a	Adakah anda **pernah**, bagi suatu tempoh, berasa diri anda sangat bersemangat atau penuh bertenaga sehingga anda mengalami kesulitan, atau orang lain berpendapat bahawa anda bukan diri anda yang biasanya? (Jangan masukkan waktu di mana anda di bawah pengaruh ubat atau alkohol)<br>JIKA PESAKIT BINGUNG ATAU TIDAK JELAS TENTANG APA YANG DIMAKSUDKAN DENGAN "BERSEMANGAT" ATAU "PENUH TENAGA", JELASKAN SEPERTI BERIKUT: Maksud "bersemangat" atau "penuh tenaga" ialah: perasaan yang sangat gembira, tenaga yang bertambah, memerlukan sedikit tidur, jalan fikiran menjadi cepat, mempunyai banyak idea; produktiviti, kreativiti, motivasi atau tingkah laku impulsif meningkat.	TIDAK	YA	1
b	JIKA YA:<br>Adakah anda kini berasa sangat bersemangat atau penuh bertenaga?	TIDAK	YA	2
D2a	Adakah anda pernah tersinggung secara berterusan, untuk beberapa hari, sehingga anda berbalah, bertengkar atau berkelahi, atau berteriak pada orang di luar keluarga anda? Adakah anda atau orang lain memerhatikan bahawa anda lebih mudah tersinggung atau bereaksi berlebihan, dibandingkan dengan orang lain, meskipun dalam keadaan yang menurut anda beralasan? (Jangan masukkan waktu di mana anda di bawah pengaruh ubat atau alkohol)	TIDAK	YA	3
b	JIKA YA:<br>Adakah anda kini berasa tersinggung secara berterusan?	TIDAK	YA	4
	ADAKAH **D1a** ATAU **D2a** DIBERI KOD YA?	→ TIDAK	YA	

D3 JIKA D1b ATAU D2b = YA : PERIKSA HANYA EPISOD KINI
JIKA D1b DAN D2b = TIDAK : PERIKSA EPISOD LALU YANG PALING SIMTOMATIK

Pada waktu anda berasa sangat bersemangat, penuh bertenaga dan/atau mudah tersinggung, adakah anda:

a	Berasa bahawa anda dapat melakukan apa yang orang lain tidak dapat lakukan, atau bahawa anda adalah seorang yang penting?	TIDAK	YA	
b	Hanya memerlukan sedikit tidur (misalnya, berasa segar setelah tidur hanya beberapa jam)?	TIDAK	YA	6
c	Bercakap terlalu banyak tanpa berhenti, atau bercakap terlalu cepat sehingga orang lain sukar untuk memahaminya?	TIDAK	YA	7
d	Mempunyai fikiran pantas?	TIDAK	YA	8
e	Mudah dialihkan perhatian sehingga gangguan kecil saja pun dapat mengalihkan perhatian anda?	TIDAK	YA	9
f	Menjadi begitu aktif atau tidak berehat sehingga orang lain bimbang akan diri anda?	TIDAK	YA	10

→ BERERTI : PERGI KE KOTAK(-KOTAK) DIAGNOSTIK DARI MODUL INI, BULATKAN TIDAK DALAM SEMUA KOTAK DAN PINDAH KE MODUL BERIKUTNYA

g Sangat ingin melakukan aktiviti yang menyeronokkan sehingga anda tidak menghiraukan risiko atau akibatnya (misalnya, belanja berlebihan, memandu secara semberono, atau kelakuan seksual yang tidak berhemat)? TIDAK YA 11

ADAKAH 3 ATAU LEBIH JAWAPAN D3 DIBERI KOD YA ATAU 4 JIKA D1a = TIDAK (EPISOD LALU) ATAU D1b = TIDAK (EPISOD KINI)? → TIDAK YA

D4 Adakah gejala itu berlanjutan sekurang-kurangnya satu minggu dan menyebabkan masalah yang ketara di rumah, tempat kerja, atau sekolah, atau adakah anda dimasukkan ke dalam hospital atas masalah ini? TIDAK YA 12

JIKA YA UNTUK SALAH SATU, BERI KOD YA

ADAKAH D4 DIBERI KOD TIDAK?

JIKA YA, TENTUKAN SAMA ADA EPISOD ITU IALAH EPISOD KINI ATAU EPISOD LALU

TIDAK	YA
EPISOD HIPOMANIK	
KINI	☐
LALU	☐

ADAKAH D4 DIBERI KOD YA?

JIKA YA, TENTUKAN SAMA ADA EPISOD ITU IALAH EPISOD KINI ATAU EPISOD LALU

TIDAK	YA
EPISOD MANIK	
KINI	☐
LALU	☐

→ BERERTI : PERGI KE KOTAK(-KOTAK) DIAGNOSTIK DARI MODUL INI, BULATKAN TIDAK DALAM SEMUA KOTAK DAN PINDAH KE MODUL BERIKUTNYA

E. GANGGUAN PANIK

E1	Pernahkah anda, lebih dari sekali, mengalami serangan sedemikian apabila anda **tiba-tiba** berasa cemas, takut, tidak selesa atau tidak senang hati, meskipun dalam keadaan di mana kebanyakan orang tidak berasa sedemikian? Adakah serangan itu mencapai puncaknya dalam masa 10 minit?<br>BERI KOD YA HANYA APABILA SERANGAN MENCAPAI PUNCAK DALAM MASA 10 MINIT	TIDAK YA	1
	JIKA E1 = TIDAK, BULATKAN TIDAK PADA E5 DAN LANGKAU KE F1		
E2	Pada masa lalu, adakah serangan itu datang secara tidak dijangka, atau terjadi tanpa diduga atau dibangkitkan?<br>JIKA E2 = TIDAK, BULATKAN TIDAK PADA E5 DAN LANGKAU KE F1	TIDAK YA	2
E3	Adakah anda pernah mengalami serangan sedemikian dan kemudian bimbang akan kejadian satu serangan lagi, atau bimbang akan akibat serangan itu dalam sebulan yang berikutnya atau lebih?<br>JIKA E3 = TIDAK, BULATKAN TIDAK PADA E5 DAN LANGKAU KE F1	TIDAK YA	3
E4	**Menjelang serangan terburuk yang anda ingat:**		
a	Adakah denyutan jantung anda tak teratur, cepat atau berdegap-degap?	TIDAK YA	4
b	Adakah tangan anda berpeluh atau berlengas?	TIDAK YA	5
c	Adakah anda gementar atau bergetar?	TIDAK YA	6
d	Adakah anda berasa sesak nafas atau menghadapi kesulitan untuk bernafas?	TIDAK YA	7
e	Adakah anda berasa tercekik atau tersumbat di kerongkong?	TIDAK YA	8
f	Adakah anda berasa sakit, tertekan atau tidak selesa di dada?	TIDAK YA	9
g	Adakah anda berasa loya, masalah perut atau cirit-birit secara mendadak?	TIDAK YA	10
h	Adakah anda berasa pening, tidak stabil atau pengsan?	TIDAK YA	11
i	Adakah anda berasa aneh, tidak nyata, terasing atau tidak kenal akan sekeliling anda; atau anda berasa berada di luar atau terpisah dari sebahagian atau keseluruhan badan anda?	TIDAK YA	12
j	Adakah anda takut hilang kawalan atau menjadi gila?	TIDAK YA	13
k	Adakah anda berasa akan mati?	TIDAK YA	14
l	Adakah anda berasa bahagian badan anda berdenyut-denyut atau kebas?	TIDAK YA	15
m	Adakah anda mengalami kilatan panas atau kesejukan?	TIDAK YA	16
E5	ADAKAH 4 ATAU LEBIH JAWAPAN E4 DIBERI KOD YA?<br>JIKA E5 = TIDAK, LANGKAU KE E7	TIDAK YA<br>*Gangguan Panik Seumur hidup*	
E6	Dalam sebulan yang lalu, adakah anda mengalami serangan secara berulang kali (2 atau lebih) diikuti oleh kebimbangan berterusan terhadap kejadian satu serangan lagi?<br>JIKA E6 = YA, LANGKAU KE F1	TIDAK YA<br>*Gangguan Panik Kini*	17
E7	ADAKAH 1, 2 atau 3 JAWAPAN E4 DIBERI KOD YA ?	TIDAK YA<br>*Serangan dengan gejala terhad Seumur hidup*	18

M.I.N.I. 5.0.0 **Malay version / DSM-IV / current (August 1998)**
Y. Lecrubier, E. Weiller, T. Hergueta, P. Amorim, L.I. Bonora, J.P. Lépine (INSERM-PARIS, FRANCE) / D. Sheehan, J. Janavs, R. Baker, K.H. Sheehan, E. Knapp, M. Sheehan (University of South Florida-TAMPA, USA) Translation into Malay, December 2003

F. AGORAFOBIA

F1 Adakah anda berasa cemas atau tidak senang hati di tempat atau keadaan di mana anda akan diserang perasaan panik atau gejala seperti panik yang disebut tadi, atau mungkin tiada pertolongan atau anda tidak dapat menjauhkan diri: seperti berada di tengah orang ramai, beratur, apabila anda bersendirian dan jauh dari rumah atau bersendirian di rumah, atau semasa menyeberangi jambatan, menaiki bus, kereta api, atau kereta? TIDAK YA 19

JIKA F1 = TIDAK, BULATKAN TIDAK PADA F2

F2 Adakah anda sangat takut akan keadaan tersebut sehingga anda mengelakkannya, atau anda menderita, atau memerlukan teman untuk menghadapi keadaan itu? TIDAK YA *Agorafobia Kini*

ADAKAH F2 (AGORAFOBIA KINI) DIBERI KOD **TIDAK**
dan
ADAKAH E6 (GANGGUAN PANIK KINI) DIBERI KOD **YA**?

TIDAK YA
GANGGUAN PANIK tanpa Agorafobia KINI

ADAKAH F2 (AGORAFOBIA KINI) DIBERI KOD **YA**
dan
ADAKAH E6 (GANGGUAN PANIK KINI) DIBERI KOD **YA**?

TIDAK YA
GANGGUAN PANIK dengan Agorafobia KINI

ADAKAH F2 (AGORAFOBIA KINI) DIBERI KOD **YA**
dan
ADAKAH E5 (GANGGUAN PANIK SEUMUR HIDUP) DIBERI KOD **TIDAK**?

TIDAK YA
AGORAFOBIA Tanpa sejarah Gangguan Panik KINI

F. AGORAFOBIA

F1 Adakah anda berasa cemas atau tidak senang hati di tempat atau keadaan di mana anda akan diserang perasaan panik atau gejala seperti panik yang disebut tadi, atau mungkin tiada pertolongan atau anda tidak dapat menjauhkan diri: seperti berada di tengah orang ramai, beratur, apabila anda bersendirian dan jauh dari rumah atau bersendirian di rumah, atau semasa menyeberangi jambatan, menaiki bus, kereta api, atau kereta? TIDAK YA 19

JIKA F1 = TIDAK, BULATKAN TIDAK PADA F2

F2 Adakah anda sangat takut akan keadaan tersebut sehingga anda mengelakkannya, atau anda menderita, atau memerlukan teman untuk menghadapi keadaan itu? TIDAK YA *Agorafobia Kini*

ADAKAH F2 (AGORAFOBIA KINI) DIBERI KOD **TIDAK**
dan
ADAKAH E6 (GANGGUAN PANIK KINI) DIBERI KOD **YA**?

TIDAK	YA
GANGGUAN PANIK tanpa Agorafobia KINI	

ADAKAH F2 (AGORAFOBIA KINI) DIBERI KOD **YA**
dan
ADAKAH E6 (GANGGUAN PANIK KINI) DIBERI KOD **YA**?

TIDAK	YA
GANGGUAN PANIK dengan Agorafobia KINI	

ADAKAH F2 (AGORAFOBIA KINI) DIBERI KOD **YA**
dan
ADAKAH E5 (GANGGUAN PANIK SEUMUR HIDUP) DIBERI KOD **TIDAK**?

TIDAK	YA
AGORAFOBIA Tanpa sejarah Gangguan Panik KINI	

H. GANGGUAN OBSESI-KOMPULSI

H1	Dalam bulan yang lalu, adakah anda diresahkan oleh fikiran, dorongan atau bayangan lalu yang tidak diingini, tidak menyenangkan, tidak wajar, mendesak atau menekan? (misalnya fikiran bahawa diri anda adalah kotor, tercemar, atau mempunyai kuman, **atau** takut mencemari orang lain, **atau** takut mencederakan seseorang walaupun anda tidak inginkannya, atau takut anda akan bertindak mengikuti dorongan tertentu, **atau** takut atau mempunyai kepercayaan karut bahawa anda bertanggungjawab atas hal-hal yang salah, **atau** penuh dengan fikiran, bayangan atau dorongan seksual, **atau** mengumpulkan barang-barang, **atau** banyak berfikir tentang agama.)	TIDAK	YA	1

JANGAN MASUKKAN KEBIMBANGAN BERLEBIHAN TERHADAP MASALAH DALAM HIDUP NYATA. JANGAN MASUKKAN OBSESI YANG BERKAITAN DENGAN GANGGUAN MAKAN, PENYIMPANGAN SEKSUAL, PERJUDIAN PATOLOGIK, ATAU PENYALAHGUNAAN ALKOHOL ATAU UBAT, KERANA PESAKIT DAPAT MENIKMATI KESERONOKAN DARI AKTIVITI TERSEBUT DAN MUNGKIN INGIN MELAWANNYA ATAS SEBAB AKIBAT NEGATIF YANG ADA.

JIKA H1 = TIDAK, LANGKAU KE H4

H2	Adakah hal itu tetap datang kembali ke dalam fikiran anda meskipun anda cuba tidak menghiraukan atau berusaha menghilangkan hal itu?	TIDAK	YA	2

JIKA H2 = TIDAK, LANGKAU KE H4

H3	Adakah anda berpendapat bahawa obsesi ini merupakan hasil fikiran anda dan bukan dikenakan dari luar?	TIDAK	YA	3
H4	Dalam bulan yang lalu, adakah anda melakukan sesuatu berulang kali tanpa dapat menahannya, seperti mencuci atau membersihkan secara berlebihan, mengira atau memeriksa berulang kali, atau mengulangi, mengumpulkan, mengatur barang-barang, atau menjalankan upacara kepercayaan karut?	TIDAK	YA	4

ADAKAH H3 <u>ATAU</u> H4 DIBERI KOD YA? → TIDAK YA

H5	Adakah anda menyedari bahawa fikiran obsesi dan/atau tingkah laku kompulsi anda berlebihan atau tidak masuk akal?	→ TIDAK	YA	5
H6	Adakah fikiran obsesi dan/atau tingkah laku kompulsi ini mengganggu kegiatan harian, pekerjaan, aktiviti atau hubungan sosial anda, atau menghabiskan waktu lebih dari satu jam sehari?	TIDAK	YA	6

ADAKAH H6 DBERI KOD YA?

TIDAK	YA
GANGGUAN OBSESI-KOMPULSI KINI	

→ BERERTI : PERGI KE KOTAK(-KOTAK) DIAGNOSTIK DARI MODUL INI, BULATKAN TIDAK DALAM SEMUA KOTAK DAN PINDAH KE MODUL BERIKUTNYA

I. GANGGUAN TEKANAN SELEPAS TRAUMA (secara pilihan)

I1 Adakah anda pernah mengalami, atau menyaksikan, atau menghadapi peristiwa yang sangat traumatik termasuk kematian nyata atau ancaman kematian atau cedera yang parah terhadap anda atau orang lain? → TIDAK YA 1

CONTOH PERISTIWA TRAUMATIK: KECELAKAAN SERIUS, SERANGAN SEKSUAL ATAU FIZIKAL, SERANGAN PENGGANAS, DIJADIKAN ORANG TEBUSAN, PENCULIKAN, KEBAKARAN, MENEMUI MAYAT, KEMATIAN YANG TIDAK TERDUGA, PERANG, BENCANA ALAM...

I2 Dalam bulan yang lalu, adakah anda mengalami semula peristiwa tersebut dengan cara yang menimbulkan tekanan (misalnya, mimpi, ingatan kuat, ulang kenang, atau reaksi fizikal)? → TIDAK YA 2

I3 **Dalam bulan yang lalu:**

a Adakah anda mengelak berfikir atau bercakap tentang peristiwa itu, atau adakah anda mengelak hal-hal yang mengingatkan anda akan peristiwa itu? TIDAK YA 3

b Adakah anda menghadapi kesulitan mengingat kembali bahagian-bahagian penting dalam kejadian itu? TIDAK YA 4

c Adakah anda menjadi kurang berminat dalam hobi atau aktiviti sosial? TIDAK YA 5

d Adakah anda berasa terpisah atau terasing dari orang lain? TIDAK YA 6

e Adakah anda menyedari bahawa perasaan anda menjadi kaku? TIDAK YA 7

f Adakah anda berasa bahawa hayat anda akan lebih pendek akibat daripada trauma tersebut? TIDAK YA 8

ADAKAH 3 ATAU LEBIH JAWAPAN I3 DIBERI KOD YA? → TIDAK YA

I4 **Dalam bulan yang lalu:**

a Adakah anda menghadapi kesulitan untuk tidur? TIDAK YA 9

b Adakah anda mudah tersinggung atau adakah kemarahan anda tiba-tiba meledak? TIDAK YA 10

c Adakah anda menghadapi kesulitan untuk menumpukan perhatian? TIDAK YA 11

d Adakah anda berasa cemas atau sentiasa berjaga-jaga? TIDAK YA 12

e Adakah anda mudah terkejut? TIDAK YA 1[illegible]

ADAKAH 2 ATAU LEBIH JAWAPAN I4 DIBERI KOD YA? → TIDAK YA

I5 Dalam bulan yang lalu, adakah masalah ini secara ketara mengganggu perkerjaan, aktiviti sosial anda atau menyebabkan tekanan yang ketara? TIDAK YA 14

ADAKAH I5 DIBERI KOD YA?

TIDAK	YA
GANGGUAN TEKANAN SELEPAS TRAUMA KINI	

→ BERERTI : PERGI KE KOTAK(-KOTAK) DIAGNOSTIK DARI MODUL INI, BULATKAN TIDAK DALAM SEMUA KOTAK DAN PINDAH KE MODUL BERIKUTNYA

J. PENYALAHGUNAAN DAN PERGANTUNGAN KEPADA ALKOHOL

J1 Dalam 12 bulan yang lalu, adakah anda minum 3 atau lebih minuman beralkohol dalam masa 3 jam dalam 3 atau lebih kesempatan? → TIDAK YA 1

J2 **Dalam 12 bulan yang lalu:**

a Adakah anda perlu minum lebih banyak untuk mendapatkan kesan sama yang anda dapatkan apabila mula minum? TIDAK YA 2

b Apabila anda mengurangkan jumlah minuman, adakah tangan anda bergetar, adakah anda berpeluh, atau berasa gelisah?
Atau, adakah anda minum untuk mengelakkan gejala-gejala itu atau mengelakkan *hangover* (kesan-kesan selepas terlalu banyak minum), misalnya gementar, berpeluh atau gelisah?
JIKA YA UNTUK SALAH SATU, BERI KOD YA TIDAK YA 3

c Apabila anda minum alkohol, adakah anda akhirnya minum lebih banyak daripada yang dirancangkan? TIDAK YA 4

d Adakah anda cuba mengurangkan atau berhenti minum alkohol tetapi gagal? TIDAK YA 5

e Pada hari-hari anda minum, adakah anda menghabiskan kebanyakan masa untuk mendapatkan alkohol, minum, atau untuk pulih dari kesan alkohol? TIDAK YA 6

f Adakah anda menghabiskan sedikit masa untuk bekerja, menikmati hobi, atau bersama orang lain akibat daripada tabiat minum ini? TIDAK YA 7

g Adakah anda terus minum meskipun anda tahu bahawa minum itu membawa masalah kesihatan atau mental kepada diri anda? TIDAK YA 8

ADAKAH 3 ATAU LEBIH JAWAPAN J2 DIBERI KOD YA?

TIDAK	YA
PERGANTUNGAN KEPADA ALKOHOL KINI	

ADAKAH PESAKIT MENDAPAT KOD POSITIF UNTUK PERGANTUNGAN KEPADA ALKOHOL? TIDAK YA →

J3 **Dalam 12 bulan yang lalu:**

a Adakah anda pernah mengalami mabuk atau *hangover* (kesan-kesan selepas terlalu banyak minum) lebih daripada satu kali apabila anda memikul tanggungjawab di sekolah, tempat kerja, atau rumah? Adakah hal ini menimbulkan masalah?
BERI KOD YA HANYA APABILA HAL INI MENIMBULKAN MASALAH TIDAK YA 9

b Adakah anda mabuk lebih daripada satu kali dalam keadaan yang berisiko untuk diri anda, misalnya memandu kereta, menunggang motosikal, menggunakan mesin, berlayar, dll.? TIDAK YA 10

→ BERERTI : PERGI KE KOTAK(-KOTAK) DIAGNOSTIK DARI MODUL INI, BULATKAN TIDAK DALAM SEMUA KOTAK DAN PINDAH KE MODUL BERIKUTNYA

c	Adakah anda menghadapi masalah undang-undang lebih daripada satu kali akibat daripada minum anda, misalnya ditahan atau melanggar undang-undang?	TIDAK	YA	11
d	Adakah anda terus minum meskipun tabiat minum anda menimbulkan masalah dengan keluarga dan orang lain?	TIDAK	YA	12

ADAKAH 1 ATAU LEBIH JAWAPAN J3 DIBERI KOD YA?

TIDAK	YA
	PENYALAHGUNAAN ALKOHOL KINI

CARD OF SUBSTANCES

AMFETAMIN	BENSIN	CEU IIN
CIMENG	DOBEL L / LELE	ECSTASY / INEX
ETHER	GANJA	GELEK
HASIS	HEROIN	KODEIN
KOKAIN	LEM	LSD
MARIJUANA	METHADONE	MG / BK
MORFIN	MUSHROOM	OPIUM
PCP	PIL KOPLO	PUTAW
RITALIN	RUMPUT	THC

M.I.N.I.

K. GANGGUAN PENGGUNAAN BAHAN PSIKOAKTIF BUKAN ALKOHOL

K1 Sekarang saya akan tunjukkan kepada anda (TUNJUKKAN CARTA BAHAN) / saya akan bacakan kepada anda (BACAKAN SENARAI DI BAWAH INI) senarai ubat. Dalam 12 bulan yang lalu, adakah anda pernah mengambil ubat-ubat ini, lebih daripada sekali, untuk menikmatinya, berasa lebih baik, atau untuk mengubah angin anda? → TIDAK YA

BULATKAN SE TIAP UBAT YANG DIAMBIL:

Perangsang: amfetamin, « *sabu* », « *speed* », kristal *meth*, « *rush* », Dexedrine, Ritalin, pil diet.
Kokain: berdengus, IV, *freebase*, *crack*, « *speedball* ».
Narkotik: heroin, morfin, opium, methadone, kodein, petidin.
Halusinogen: LSD (« *asid* »), meskalin, peyote, PCP (« *angel dust* », « *peace pill* »), psilosibin, STP, « *mushroom* », ecstasy, MDA, or MDMA.
Penyedut: « perekat », bensin, *ethyl chloride*, *nitrous oxide*, (« gas gelak »), amyl atau butyl nitrate (« *poppers* »).
Marijuana: hashish (« hash »), THC, « pot », « rumput », « rumput laut », « rokok marijuana ».
Penenang: « pil koplo », « BK », « MG », Valium, Xanax, Librium, Ativan, Halcion, barbiturates.
Lain-lain: trihexyfenidil (« *double L* », « *lele* »), steroid, pil diet atau pil tidur yang tidak dipreskripsi. Lain-lain?

TULISKAN UBAT(-UBAT) YANG PALING SERING DIGUNAKAN: ______________________

TENTUKAN MANA YANG AKAN DITANYAKAN LEBIH LANJUT DALAM KRITERIA BERIKUT:

- JIKA PENGGUNAAN BAHAN MULTIPEL BERSAMAAN ATAU BERGANTIAN:
  SETIAP UBAT (ATAU GOLONGAN UBAT) YANG DIGUNAKAN ▯
  UBAT (ATAU GOLONGAN UBAT) YANG PALING BANYAK DIGUNAKAN ▯
- JIKA SATU UBAT (ATAU GOLONGAN UBAT) YANG DIGUNAKAN:
  UBAT (ATAU GOLONGAN UBAT) TUNGGAL ▯

K2 **Mempertimbangkan anda menggunakan [SEBUTKAN UBAT/GOLONGAN UBAT YANG DIPILIH] dalam 12 bulan yang lalu:**

a Adakah anda mendapati bahawa anda harus menggunakan lebih banyak [SEBUTKAN UBAT/GOLONGAN UBAT YANG DIPILIH] untuk mendapatkan kesan sama yang anda dapatkan apabila mula menggunakannya? TIDAK YA 1

b Apabila anda mengurangkan atau menghentikan penggunaan [SEBUTKAN UBAT/GOLONGAN UBAT YANG DIPILIH], adakah anda mengalami gejala melepaskan ubat (sakit, gementar, demam, rasa lemah, cirit-birit, rasa loya, berpeluh, hati berdegap-degap, kesulitan untuk tidur, atau rasa gelisah, cemas, mudah tersinggung atau tertekan)?
Atau adakah anda menggunakan sebarang ubat untuk mengelakkan diri dari gejala melepaskan ubat atau agar berasa lebih baik? TIDAK YA 2
JIKA **YA** UNTUK SALAH SATU, BERI KOD **YA**

c Adakah anda sering mendapati bahawa jika anda menggunakan [SEBUTKAN UBAT/GOLONGAN UBAT YANG DIPILIH] anda akhirnya mengambil lebih banyak dari yang anda fikirkan? TIDAK YA 3

d Adakah anda pernah cuba mengurangkan atau menghentikan penggunaan [SEBUTKAN UBAT/GOLONGAN UBAT YANG DIPILIH] tetapi gagal? TIDAK YA 4

e	Pada hari-hari anda menggunakan [SEBUTKAN UBAT/GOLONGAN UBAT YANG DIPILIH], adakah anda menghabiskan kebanyakan masa (>2 jam) untuk mendapatkan, menggunakan atau untuk pulih dari kesan ubat, atau memikirkan tentangnya?	TIDAK	YA	5
f	Adakah anda menghabiskan sedikit masa untuk bekerja, menikmati hobi, atau bersama keluarga atau kawan akibat daripada penggunaan ubat?	TIDAK	YA	6
g	Adakah anda melanjutkan penggunaan [SEBUTKAN UBAT/GOLONGAN UBAT YANG DIPILIH] meskipun ia menimbulkan masalah kesihatan atau mental kepada diri anda?	TIDAK	YA	7

ADAKAH 3 ATAU LEBIH JAWAPAN K2 DIBERI KOD YA?

SEBUTKAN UBAT(-UBAT): ______________________

TIDAK	YA
PERGANTUNGAN KEPADA UBAT KINI	

ADAKAH PESAKIT MENDAPAT KOD POSITIF UNTUK PERGANTUNGAN KEPADA UBAT? → TIDAK YA

K3 **Dalam 12 bulan yang lalu:**

a	Adakah anda pernah mengalami mabuk atau *hangover* akibat daripada [SEBUTKAN UBAT/GOLONGAN UBAT YANG DIPILIH] lebih dari satu kali apabila anda memikul tanggungjawab di sekolah, tempat kerja, atau rumah? Adakah hal ini menimbulkan masalah? (BERI KOD YA HANYA APABILA HAL INI MENIMBULKAN MASALAH)	TIDAK	YA	8
b	Adakah anda pernah mengalami mabuk atau di bawah pengaruh [SEBUTKAN UBAT/GOLONGAN UBAT YANG DIPILIH] dalam keadaan yang berisiko untuk diri anda (misalnya memandu kereta, menunggang motosikal, menggunakan mesin, berlayar, dll.)?	TIDAK	YA	9
c	Adakah anda menghadapi masalah undang-undang lebih daripada satu kali akibat daripada penggunaan [SEBUTKAN UBAT/GOLONGAN UBAT YANG DIPILIH], misalnya ditahan atau melanggar undang-undang?	TIDAK	YA	10
d	Adakah anda terus menggunakan [SEBUTKAN UBAT/GOLONGAN UBAT YANG DIPILIH] meskipun hal itu menimbulkan masalah dengan keluarga dan orang lain?	TIDAK	YA	11

ADAKAH 1 ATAU LEBIH JAWAPAN K3 DIBERI KOD YA?

SEBUTKAN UBAT(-UBAT): ______________________

TIDAK	YA
PENYALAHGUNAAN UBAT KINI	

L. GANGGUAN PSIKOTIK

MINTALAH CONTOH UNTUK SETIAP PERTANYAAN YANG DIJAWAB SECARA POSITIF. BERILAH KOD YA HANYA APABILA CONTOH TERSEBUT JELAS MENUNJUKKAN GANGGUAN FIKIRAN ATAU TANGGAPAN ATAU JIKA HAL-HAL ITU TIDAK WAJAR DARI SEGI BUDAYA.
SEBELUM MEMBERI KOD, PERIKSALAH SAMA ADA DELUSI DAPAT DIGOLONGKAN SEBAGAI « ANEH ».

DELUSI DIANGGAP ANEH APABILA: BENAR-BENAR TIDAK BOLEH DIPERCAYAI, TIDAK MASUK AKAL, TIDAK DAPAT DIFAHAMI, DAN TIDAK DAPAT DIPEROLEHI DARI PENGALAMAN HIDUP HARIAN.

HALUSINASI DIANGGAP ANEH APABILA: SUATU SUARA MEMBERI KOMEN ATAS FIKIRAN ATAU TINGKAH LAKU ORANG TERSEBUT; ATAU ADA DUA ATAU LEBIH SUARA YANG SALING BERCAKAP-CAKAP.

Sekarang saya akan bertanya kepada anda tentang pengalaman yang tidak lazim yang mungkin dialami orang lain.

				ANEH	
L1 a	Adakah anda pernah percaya bahawa ada orang mengintip anda, atau bahawa ada orang berkomplot terhadap anda, atau cuba menyakiti anda?	TIDAK	YA	YA	1
b	JIKA YA: Adakah anda sekarang percaya akan hal ini?	TIDAK	YA	YA → L6a	2
L2 a	Adakah anda pernah percaya bahawa seseorang sedang membaca fikiran anda atau dapat mendengar fikiran anda atau bahawa anda dapat membaca atau mendengar apa yang sedang difikirkan orang lain?	TIDAK		YA	3
b	JIKA YA: Adakah anda sekarang percaya akan hal ini?	TIDAK		YA → L6a	4
L3 a	Adakah anda pernah percaya bahawa seseorang atau suatu kuasa di luar diri anda memasukkan gagasan ke dalam fikiran anda, atau membuat anda bertindak di luar diri anda yang biasa? Adakah anda pernah percaya bahawa anda kerasukan?	TIDAK		YA	5
b	JIKA YA: Adakah anda sekarang percaya akan hal ini?	TIDAK		YA → L6a	6
L4 a	Adakah anda pernah percaya bahawa anda dikirimi mesej khas melalui TV, radio atau suratkhabar, atau bahawa seseorang yang tidak dikenali menaruh minat terhadap anda?	TIDAK	YA	YA	7
b	JIKA YA: Adakah anda sekarang percaya akan hal ini?	TIDAK	YA	YA → L6a	8
L5 a	Adakah saudara-mara atau kawan anda pernah menganggap kepercayaan anda aneh atau tidak sesuai dengan kenyataan? DELUSI LAIN YANG TIDAK DIPERIKSA DALAM PERTANYAAN L1 KE L4, MISALNYA TENTANG KEBESARAN, PERASAAN IRI HATI, RASA BERSALAH, KEHANCURAN ATAU KEMISKINAN...	TIDAK	YA	YA	9
b	JIKA YA: Adakah mereka sekarang menganggap kepercayaan anda aneh?	TIDAK	YA	YA	10
L6 a	Adakah anda pernah mendengar suara yang tidak dapat didengari orang lain, seperti suara orang? HALUSINASI DIANGGAP « ANEH » HANYA APABILA PESAKIT MENJAWAB YA UNTUK PERTANYAAN YANG BERIKUT: Adakah anda mendengar suara orang memberi komen atas fikiran atau tingkah laku anda, atau adakah anda mendengar dua atau lebih suara yang saling bercakap?	TIDAK	YA	YA	11
b	JIKA YA: Adakah anda mendengar hal-hal ini dalam bulan yang lalu?	TIDAK	YA	YA → L8b	12

M.I.N.I. 5.0.0 Malay version / DSM-IV / current (August 1998)
Y. Lecrubier, E. Weiller, T. Hergueta, P. Amorim, L.I. Bonora, J.P. Lepine (INSERM-PARIS, FRANCE) / D. Sheehan, J. Janavs, R. Baker, K.H. Sheehan, E. Knapp, M. Sheehan (University of South Florida-TAMPA, USA) Translation into Malay, December 2003

L7 a Adakah anda pernah melihat bayangan sewaktu anda bangun (tidak tidur) atau pernahkan anda melihat hal-hal yang tidak dapat dilihat oleh orang lain? TIDAK YA 13

BERI KOD YA HANYA APABILA PENGLIHATAN ITU ADALAH TIDAK WAJAR DARI SEGI BUDAYA.

b JIKA YA: Adakah anda melihat hal-hal ini dalam bulan yang lalu? TIDAK YA 14

PERTIMBANGAN PEWAWANCARA:

L8 b ADAKAH PESAKIT SEKARANG MENUNJUKKAN PERCAKAPAN YANG TIDAK KERUAN, TIDAK TERATUR ATAU TIDAK TEPAT? TIDAK YA 15

L9 b ADAKAH PESAKIT SEKARANG MENUNJUKKAN TINGKAH LAKU YANG TIDAK TERATUR ATAU KATATONIK? TIDAK YA 16

L10b ADAKAH GEJALA NEGATIF SKIZOFRENIA, SEPERTI KESAN YANG DATAR, KEKURANGAN PERCAKAPAN (ALOGIA) ATAU KETIDAKUPAYAAN MEMULAKAN ATAU MEMPERTAHANKAN AKTIVITI BERTUJUAN (AVOLISI), TERLIHAT MENJELANG WAWANCARA? TIDAK YA 17

L11 DARI L1 HINGGA L10:
- ADAKAH 1 ATAU LEBIH PERTANYAAN « b » DIBERI KOD YA ANEH?

ATAU
- ADAKAH 2 ATAU LEBIH PERTANYAAN « b » DIBERI KOD YA (BUKAN YA ANEH)?

TIDAK YA

GANGGUAN PSIKOTIK KINI

L12 DARI L1 HINGGA L7:
- ADAKAH 1 ATAU LEBIH PERTANYAAN « a » DIBERI KOD YA ANEH?

ATAU
- ADAKAH 2 ATAU LEBIH PERTANYAAN « a » DIBERI KOD YA (BUKAN YA ANEH)?

(PERIKSA SAMA ADA 2 GEJALA ITU TERJADI PADA TEMPOH YANG SAMA)

ATAU
- ADAKAH L11 DIBERI KOD YA?

TIDAK YA

SINDROM PSIKOTIK SEUMUR HIDUP

L13a JIKA L12 DIBERI KOD YA ATAU SEKURANG-KURANGNYA SATU YA DARIPADA L1 HINGGA L7:

ADAKAH PESAKIT MENDAPAT KOD POSITIF UNTUK:
EPISOD TEKANAN UTAMA (KINI ATAU LALU)
ATAU EPISOD MANIK (KINI ATAU LALU)? → TIDAK YA

b Anda katakan sebelum ini anda pernah berasa tertekan/bersemangat/mudah tersinggung bagi suatu tempoh. Adakah kepercayaan dan pengalaman yang baru anda gambarkan (GEJALA YANG DIBERI KOD YA DARI L1 HINGGA L7) hanya terhad kepada waktu anda berasa tertekan/bersemangat/mudah tersinggung? TIDAK YA 18

ADAKAH L13b DIBERI KOD YA?

TIDAK YA

GANGGUAN ANGIN DENGAN GAMBARAN PSIKOTIK KINI

N. BULIMIA NERVOSA

N1 Dalam tiga bulan yang lalu, adakah anda makan mengikut nafsu atau anda makan sejumlah besar makanan dalam waktu 2 jam? → TIDAK YA 8

N2 Dalam 3 bulan yang lalu, adakah anda makan mengikut nafsu sehingga dua kali seminggu? → TIDAK YA 9

N3 Apabila anda makan mengikut nafsu, adakah anda berasa bahawa makan anda tidak terkawal? → TIDAK YA 10

N4 Adakah anda melakukan sesuatu untuk mengimbangi, atau untuk mencegah bertambahnya berat badan akibat daripada makan mengikut nafsu, seperti muntah, puasa, bersenam atau mengambil julap, ubat diet, atau ubat lain? → TIDAK YA 11

N5 Adakah berat badan atau bentuk badan anda sangat mempengaruhi perasaan anda terhadap diri sendiri? → TIDAK YA 12

N6 ADAKAH GEJALA-GEJALA PESAKIT MEMENUHI KRITERIA UNTUK ANOREXIA NERVOSA? TIDAK YA 13

JIKA N6 = TIDAK, LANGKAU KE N8

N7 Adakah makan mengikut nafsu hanya terjadi apabila berat badan anda di bawah ______kg*? TIDAK YA 14

* GUNAKANLAH AMBANG BERAT BADAN UNTUK PESAKIT INI DARI JADUAL TINGGI/BERAT DALAM MODUL ANOREXIA NERVOSA

N8 ADAKAH N5 DIBERI KOD YA DAN N7 DIBERI KOD TIDAK (ATAU DILANGKAUI)?

TIDAK	YA
BULIMIA NERVOSA KINI	

ADAKAH N7 DIBERI KOD YA?

TIDAK	YA
ANOREXIA NERVOSA Jenis makan mengikut nafsu /mencahar KINI	

Y. Lecrubier, E. Weiller, T. Hergueta, P. Amorim, L.I. Bonora, J.P. Lépine (INSERM-PARIS, FRANCE) / D. Sheehan, J. Janavs, R. Baker, K.H. Sheehan, E. Knapp, M. Sheehan (University of South Florida-TAMPA, USA) Translation into Malay, December 2003

O. GANGGUAN KEBIMBANGAN MENYELURUH

O1	a	Adakah anda berasa amat bimbang atau cemas akan banyak hal dalam kehidupan harian, di rumah, di tempat kerja, di dalam lingkungan anda selama 6 bulan yang lalu?<br><br>JANGAN BERI KOD YA JIKA FOKUS KEBIMBANGANNYA TERHAD KEPADA GANGGUAN LAIN YANG TELAH DIPERIKSA SEBELUM INI, SEPERTI MENDAPAT SERANGAN PANIK (GANGGUAN PANIK), DIPERMALUKAN DI DEPAN ORANG RAMAI (FOBIA SOSIAL), DICEMARI (GOK), BERAT BADAN BERTAMBAH (ANOREXIA NERVOSA)...	→ TIDAK	YA	1
	b	Adakah kebimbangan ini wujud hampir setiap hari?	→ TIDAK	YA	2
O2		Adakah anda menghadapi kesulitan untuk mengawal kebimbangan itu atau adakah ia mengganggu keupayaan anda untuk menumpu kepada apa yang sedang anda lakukan?<br><br>DARI O3a HINGGA O3f, BERI KOD TIDAK PADA GEJALA YANG TERHAD KEPADA GANGGUAN YANG TELAH DIPERIKSA SEBELUM INI	→ TIDAK	YA	3
O3		Apabila anda berasa cemas dalam 6 bulan yang lalu, adakah anda hampir setiap hari:			
	a	Berasa gelisah, tegang atau resah?	TIDAK	YA	4
	b	Berasa tegang?	TIDAK	YA	5
	c	Berasa penat, lemah atau mudah letih?	TIDAK	YA	6
	d	Menghadapi kesulitan untuk menumpukan perhatian atau fikiran anda menjadi kosong?	TIDAK	YA	7
	e	Berasa mudah tersinggung?	TIDAK	YA	8
	f	Menghadapi kesulitan tidur (sulit untuk mula tidur, terbangun di tengah malam, terbangun pada awal pagi atau tidur berlebihan)?	TIDAK	YA	9

ADAKAH 3 ATAU LEBIH JAWAPAN O3 DIBERI KOD YA?

TIDAK	YA
GANGGUAN KEBIMBANGAN MENYELURUH KINI	

P. GANGGUAN SIKAP ANTISOSIAL (secara pilihan)

P1 Sebelum anda berumur 15 tahun, adakah anda:

a	Berulang kali ponteng sekolah atau lari dari rumah semalaman?	TIDAK	YA	1
b	Berulang kali berbohong, menipu orang lain atau mencuri?	TIDAK	YA	2
c	Mula berkelahi atau membuli, mengancam orang lain?	TIDAK	YA	3
d	Sengaja merosakkan barang-barang atau membakar?	TIDAK	YA	4
e	Sengaja mencederakan binatang atau orang?	TIDAK	YA	5
f	Memaksa seseorang melakukan hubungan seks dengan anda?	TIDAK	YA	6

ADAKAH 2 ATAU LEBIH JAWAPAN P1 DIBERI KOD YA? → TIDAK YA

P2 JANGAN BERI KOD YA BAGI TINGKAH LAKU DI BAWAH INI JIKA HAL ITU DIMOTIVASI OLEH POLITIK ATAU AGAMA

Sejak berumur 15 tahun, adakah anda:

a	Berulang kali bertindak dengan cara yang menurut orang lain tidak bertanggungjawab, misalnya tidak membayar hutang, sengaja bertindak impulsif atau sengaja tidak bekerja untuk menyara diri sendiri?	TIDAK	YA	7
b	Melakukan hal yang melanggar undang-undang meskipun anda tidak tertangkap (misalnya, memusnahkan harta benda, mencuri barang di kedai, mencuri, menjual dadah, atau melakukan kejahatan)?	TIDAK	YA	8
c.	Berulang kali berkelahi secara fizikal (termasuk berkelahi dengan pasangan atau anak)?	TIDAK	YA	9
d	Sering berbohong atau menipu orang lain untuk mendapatkan wang atau keseronokan, atau berbohong untuk bergembira?	TIDAK	YA	10
e	Membahayakan orang lain tanpa sikap peduli?	TIDAK	YA	11
f	Tidak berasa bersalah selepas menyakiti, melakukan sesuatu yang tidak patut, berbohong atau mencuri, atau selepas merosakkan harta benda?	TIDAK	YA	12

ADAKAH 3 ATAU LEBIH PERTANYAAN P2 DIBERI KOD YA?

TIDAK YA

GANGGUAN SIKAP ANTISOSIAL SEUMUR HIDUP

RUJUKAN

Lecrubier Y, Sheehan D, Weiller E, Amorim P, Bonora I, Sheehan K, Janavs J, Dunbar G. The Mini International Neuropsychiatric Interview (M.I.N.I.), a short diagnostic interview : Reliability and validity according to the CIDI. *European Psychiatry*, 1997; 12: 224-231.

Sheehan DV, Lecrubier Y, Harnett Sheehan K, Janavs J, Weiller E, Bonora LI, Keskiner A, Schinka J, Knapp E, Sheehan MF, Dunbar GC. Reliability and validity of the Mini International Neuropsychiatric Interview (M.I.N.I.) according to the SCID-P. *European Psychiatry*, 1997; 12: 232-241.

Sheehan DV, Lecrubier Y, Harnett Sheehan K, Amorim P, Janavs J, Weiller E, Hergueta T, Baker R, Dunbar G. The Mini International Neuropsychiatric Interview (M.I.N.I.), : The development and validation of a structured diagnostic psychiatric interview. *Journal of Clinical Psychiatry*, 1998; 59 [suppl 20]: 22-33.

Amorim P, Lecrubier Y, Weiller E, Hergueta T, Sheehan D. DSM-III-R Psychotic disorders: procedural validity of the Mini International Neuropsychiatric Interview (M.I.N.I.). Concordance and causes for discordance with the CIDI. *European Psychiatry*, 1998; 13: 26-34.

M.I.N.I. diperkembang bersama dalam bahasa Perancis dan Inggeris. Versi asal M.I.N.I. DSM-IV bahasa Perancis dan Inggeris telah diterjemahkan dan dapat diminta daripada penulis (lihat halaman 3). Versi ICD-10 juga disediakan dalam bahasa Perancis, Inggeris, Denmark, Thai dan Indonesia.

Languages	M.I.N.I. 4.4 and previous versions	M.I.N.I. 5.0.0 +
Afrikaans		R. Emsley, N. Keyter
Arabic		O. Osman, E Al-Radi
Basque		In preparation
Bengali		H. Banerjee, A. Banerjee
Brazilian	P. Amorim	P. Amorim
Bulgarian		L.G. Hranov
Catalan		In preparation
Czech	P. Zvolsky	P. Zvolsky
Chinese		L. Caroll, K-d Juang
Croatian		In preparation
Danish	P. Bech	P. Bech, G. Bech-Andersen, T. Schütze
Dutch Flemish	E. Griez, K. Schruers, T. Overbeek, K. Demyttenaere	I. van Vliet, H. Leroy, H. van Megen
Egyptian (Arabic)		R. Haddad, W. Naja, C. Baddoura, A. Okasha
Estonian		J. Shlik, A. Aluoja, E. Kihl
Farsi/Persian		K. Khooshabi, A. Zomorodi
Finnish	M. Heikkinen, M. Lijeström, O. Tuominen	M. Heikkinen
Filipino		B.L. Conde, A. Lao
German	I. van Denffer, M. Ackenheil, R. Dietz-Bauer	M. Ackenheil, G. Stotz, R. Dietz-Bauer, A. Vossen
Gujarati		M. Patel, B. Patel
Greek	S. Beratis	T. Calligas, S. Beratis
Hebrew	J. Zohar, Y. Sasson	R. Barda, I. Levinson
Hindi		K. Batra, S. Gambir
Hungarian	I. Bitter, J. Balazs	I. Bitter, J. Balazs
Icelandic		J. Stefanson
Indonesian		A. Maramis
Italian	P. Donda, E. Weiller, I. Bonora	L. Conti, P. Donda, A. Rossi, M. Piccinelli, M. Tansella, G. Cassano
Japanese		T. Otsobo, H. Watanabe, H. Miyaoka, K. Kamijima, J. Shinoda, K. Tanaka, Y. Okajima
Korean		H. Y. Jung et al.
Latvian	V. Janavs, J. Janavs, I. Nagobads	V. Janavs, J. Janavs
Lebanese (Arabic)		R. Haddad, W. Naja, C. Baddoura
Lithuanian		V. Danilevicute
Malaysian (Chinese)		L. Caroll, K-d Juang et al
Moroccan (Arabic)		N. Kadri, M. Agoub, S. El Gnaou
Norwegian	G. Pedersen, S. Blomhoff	K. Leiknes, S. Leganger, E. Malt, U. Malt
Polish	M. Masiak, E. Jasiak	M. Masiak, J. Przychoda
Portuguese	P. Amorim	T. Guterres, P. Levy, P. Amorim
Punjabi		A. Gahunia, S. Gambhir
Romanian	O. Driga	M.D. Gheorghe
Russian		A. Bystitsky, E. Selivra, M. Bystitsky
Serbian	I. Timotijevic	I. Timotijevic
Setswana		K. Ketlogetswe
Slovak		L. Vavrušová, J. Pečeňák, Ľ. Forgáčová
Slovenian	M. Kocmur	M. Kocmur
Spanish	L. Ferrando, J. Bobes-Garcia, J. Gibert-Rahola	L. Ferrando, L. Franco-Alfonso, M. Soto, J. Bobes, O. Soto, L. Franco, J. Gibert. Adaptation for Central and South America: G. Heinze
Swedish	M. Waern, S. Andersch, M. Humble	C. Allgulander, M. Waern, M. Humble, S. Andersch, H. Ågren
Thai		P. Kittirattanapaiboon, S. Mahatnirunkul, P. Udomrat, P. Silpakit, M. Khamwongpin, S. Srikosai.
Turkish	T. Örnek, A. Keskiner, I. Vahip	Y. Yazgan
Urdu		A. Taj, S. Gambir
Welsh		In preparation
Xhosa		D. Kaminer, I. Mbanga, N. Zungu-Dirwayi, D. Stein

Kesahan M.I.N.I. dapat dihasilkan dengan sokongan sebahagian dana dari SmithKline Beecham, the Caisse Nationale d'Assurance Maladie (701061) dan Kesatuan Eropah. Penulis berterima kasih kepada Dr Pauline Powers atas nasihatnya untuk modul Anorexia Nervosa dan Bulimia. Printed, 4 July, 2006

Soalan-soalan ini bertanyakan tentang kesan masalah-masalah ereksi (penegakan zakar) yang telah anda alami dalam kehidupan seks anda **bagi tempoh 4 minggu yang lalu**. Silakan jawab soalan-soalan ini secara jujur dan sejelas yang mungkin. Silakan jawab setiap soalan dengan menandakan pada satu kotak dengan tanda betul [✓]. Jika anda tidak pasti tentang bagaimana untuk menjawab, sila berikan jawapan yang terbaik yang anda boleh.

Dalam menjawab soalan-soalan ini, definisi-definisi berikut akan digunakan:

* **Persetubuhan**

Ditakrifkan sebagai penembusan (kemasukan) ke dalam faraj pasangan anda.

** **Aktiviti seks**

Termasuk persetubuhan, bercumbu-cumbuan, permulaan dalam perbuatan seks dan pelancapan.

*** **Pancutan mani**

Ditakrifkan sebagai pemancutan air mani daripada zakar (atau rasa seperti itu).

**** **Rangsangan seks**

Termasuk situasi seperti permainan yang memberahikan bersama pasangan anda, melihat pada gambar-gambar yang memberahikan (contohnya filem atau fotograf) dan sebagainya.

1. **Dalam tempoh 4 minggu yang lalu**, berapa kalikah anda berupaya untuk mendapat ereksi (penegakan zakar) semasa aktiviti seks**?
   Silakan tanda [✓] satu kotak sahaja.

   Tiada aktiviti seks .... ☐
   Hampir selalu atau selalu .... ☐
   Kebanyakan masa (lebih banyak daripada separuh masa) .... ☐
   Kadang-kadang (lebih kurang separuh masa) .... ☐
   Beberapa kali (sangat kurang daripada separuh masa) .... ☐
   Hampir tidak pernah atau tidak pernah .... ☐

2. **Dalam tempoh 4 minggu yang lalu** apabila anda mengalami ereksi (penegakan zakar) dengan rangsangan seks****, berapa kalikah ereksi anda cukup keras untuk penembusan ke dalam faraj?
   Silakan tanda [✓] satu kotak sahaja.

   Tiada rangsangan seks .... ☐
   Hampir selalu atau selalu .... ☐
   Kebanyakan masa (lebih banyak daripada separuh masa) .... ☐
   Kadang-kadang (lebih kurang separuh masa) .... ☐
   Beberapa kali (sangat kurang daripada separuh masa) .... ☐
   Hampir tidak pernah atau tidak pernah .... ☐

3 soalan yang berikut ialah tentang ereksi (penegakan zakar) yang mungkin anda ada alami semasa persetubuhan*.

3. **Dalam tempoh 4 minggu yang lalu** apabila anda mencuba untuk bersetubuh*, berapa kalikah zakar anda berupaya untuk menembusi (memasuki) faraj pasangan anda?
   Silakan tanda [✓] satu kotak sahaja.

   Tidak mencuba untuk bersetubuh .... ☐
   Hampir selalu atau selalu.. ☐
   Kebanyakan masa (lebih banyak daripada separuh masa) .... ☐
   Kadang-kadang (lebih kurang separuh masa)... ☐
   Beberapa kali (sangat kurang daripada separuh masa)............................. ☐
   Hampir tidak pernah atau tidak pernah .... ☐

4. **Dalam tempoh 4 minggu yang lalu** semasa persetubuhan*, **berapa kalikah** anda berupaya untuk menahan ereksi (penegakan zakar) selepas anda memasukkannya ke dalam faraj pasangan anda?
   Silakan tanda [✓] satu kotak sahaja.

   Tidak mencuba untuk bersetubuh .... ☐
   Hampir selalu atau selalu.. ☐
   Kebanyakan masa (lebih banyak daripada separuh masa) .... ☐
   Kadang-kadang (lebih kurang separuh masa)... ☐
   Beberapa kali (sangat kurang daripada separuh masa)............................. ☐
   Hampir tidak pernah atau tidak pernah .... ☐

5. **Dalam tempoh 4 minggu yang lalu** semasa persetubuhan*, **bagaimanakah sukarnya** untuk mengekalkan ereksi (penegakan zakar) untuk menghabiskan persetubuhan?
   Silakan tanda [✓] satu kotak sahaja.

   Tidak cuba untuk bersetubuh .... ☐
   Teramat sukar .... ☐
   Sangat sukar.. ☐
   Sukar .... ☐
   Sedikit sukar.. ☐
   Tidak sukar.. ☐

* **Persetubuhan**: Ditakrifkan sebagai penembusan (kemasukan) ke dalam faraj pasangan anda.
** **Aktiviti seks**: Termasuk persetubuhan, bercumbu-cumbuan, permulaan dalam perbuatan seks dan pelancapan.
*** **Pancutan mani**: Ditakrifkan sebagai pemancutan air mani daripada zakar (atau rasa seperti itu).
**** **Rangsangan seks**: Termasuk situasi seperti permainan yang memberahikan bersama pasangan anda, melihat pada gambar-gambar yang memberahikan (contohnya filem atau fotograf) dan sebagainya.

6. **<u>Dalam tempoh 4 minggu yang lalu</u>** berapa kalikah anda telah cuba bersetubuh*?
Silakan tanda [✓] satu kotak sahaja.

Tiada percubaan.. ☐
1 atau 2 kali.. ☐
3 atau 4 kali.. ☐
5 atau 6 kali.. ☐
7 hingga 10 kali.. ☐
11 atau lebih daripada 11 kali.................................... ☐

7. **<u>Dalam tempoh 4 minggu yang lalu</u>** apabila anda cuba bersetubuh* berapa kerapkah ianya memuaskan **<u>anda</u>**?
Silakan tanda [✓] satu kotak sahaja.

Tidak cuba untuk bersetubuh .... ☐
Hampir selalu atau selalu.. ☐
Kebanyakan masa (lebih banyak daripada separuh masa) .... ☐
Kadang-kadang (lebih kurang separuh masa)...................... ☐
Beberapa kali (sangat kurang daripada separuh masa).......... ☐
Hampir tidak pernah atau tidak pernah............................. ☐

8. **<u>Dalam tempoh 4 minggu yang lalu</u>** sejauh manakah anda seronok bersetubuh*?
Silakan tanda [✓] satu kotak sahaja.

Tiada persetubuhan.. ☐
Teramat seronok .... ☐
Sangat seronok.. ☐
Sederhana seronok... ☐
Tidak begitu seronok .... ☐
Tidak seronok .... ☐

* **Persetubuhan**: Ditakrifkan sebagai penembusan (kemasukan) ke dalam faraj pasangan anda.
** **Aktiviti seks**: Termasuk persetubuhan, bercumbu-cumbuan, permulaan dalam perbuatan seks dan pelancapan.
*** **Pancutan mani**: Ditakrifkan sebagai pemancutan air mani daripada zakar (atau rasa seperti itu).
**** **Rangsangan seks**: Termasuk situasi seperti permainan yang memberahikan bersama pasangan anda, melihat pada gambar-gambar yang memberahikan (contohnya filem atau fotograf) dan sebagainya.

9. **Dalam tempoh 4 minggu yang lalu** apabila anda mengalami rangsangan seks**** **atau** bersetubuh* berapa kerapkah anda mengalami pemancutan mani***?
Silakan tanda [✓] satu kotak sahaja.

- Tiada rangsangan seks atau persetubuhan .... ☐
- Hampir selalu atau selalu.. ☐
- Kebanyakan masa (lebih banyak daripada separuh masa) .... ☐
- Kadang-kadang (lebih kurang separuh masa)... ☐
- Beberapa kali (sangat kurang daripada separuh masa)............................. ☐
- Hampir tidak pernah atau tidak pernah... ☐

10. **Dalam tempoh 4 minggu yang lalu** apabila anda mengalami rangsangan seks**** **atau** bersetubuh* berapa kerapkah anda mengalami rasa puncak syahwat dengan atau tanpa pemancutan mani***?
Silakan tanda [✓] satu kotak sahaja.

- Tiada rangsangan seks atau persetubuhan .... ☐
- Hampir selalu atau selalu.. ☐
- Kebanyakan masa (lebih banyak daripada separuh masa) .... ☐
- Kadang-kadang (lebih kurang separuh masa)... ☐
- Beberapa kali (sangat kurang daripada separuh masa)............................. ☐
- Hampir tidak pernah atau tidak pernah... ☐

* **Persetubuhan**: Ditakrifkan sebagai penembusan (kemasukan) ke dalam faraj pasangan anda.
** **Aktiviti seks**: Termasuk persetubuhan, bercumbu-cumbuan, permulaan dalam perbuatan seks dan pelancapan.
*** **Pancutan mani**: Ditakrifkan sebagai pemancutan air mani daripada zakar (atau rasa seperti itu).
**** **Rangsangan seks**: Termasuk situasi seperti permainan yang memberahikan bersama pasangan anda, melihat pada gambar-gambar yang memberahikan (contohnya filem atau fotograf) dan sebagainya.

2 soalan yang berikut adalah berkaitan dengan keinginan seks. Keinginan seks boleh ditakrifkan sebagai perasaan dan ini boleh termasuk keinginan untuk mempunyai pengalaman seks (cth.: pelancapan atau persetubuhan*), memikir tentang seks, atau perasaan kecewa akibat kekurangan seks.

11. **Dalam tempoh 4 minggu yang lalu** berapa kerapkah anda mempunyai rasa **keinginan seks**?
Silakan tanda [✓] satu kotak sahaja.

Kebanyakan masa (lebih banyak daripada separuh masa) .... ☐
Hampir selalu atau selalu.. ☐
Kadang-kadang (lebih kurang separuh masa).. ☐
Beberapa kali (sangat kurang daripada separuh masa).............................. ☐
Hampir tidak pernah atau tidak pernah... ☐

12. **Dalam tempoh 4 minggu yang lalu** bagaimanakah tahap **keinginan seks** anda?
Silakan tanda [✓] satu kotak sahaja.

Sangat tinggi .... ☐
Tinggi .... ☐
Sederhana... ☐
Rendah .... ☐
Sangat rendah atau tiada langsung .... ☐

* **Persetubuhan**: Ditakrifkan sebagai penembusan (kemasukan) ke dalam faraj pasangan anda.
** **Aktiviti seks**: Termasuk persetubuhan, bercumbu-cumbuan, permulaan dalam perbuatan seks dan pelancapan.
*** **Pancutan mani**: Ditakrifkan sebagai pemancutan air mani daripada zakar (atau rasa seperti itu).
**** **Rangsangan seks**: Termasuk situasi seperti permainan yang memberahikan bersama pasangan anda, melihat pada gambar-gambar yang memberahikan (contohnya filem atau fotograf) dan sebagainya.

13. **Dalam tempoh 4 minggu yang lalu** bagaimanakah kepuasan anda dengan keseluruhan **kehidupan seks** anda?
Silakan tanda [✓] satu kotak sahaja.

Sangat puas .... ☐
Sederhana puas .... ☐
Puas dan tidak puas adalah hampir sama .... ☐
Sederhana tidak puas .... ☐
Sangat tidak puas .... ☐

14. **Dalam tempoh 4 minggu yang lalu** bagaimanakah kepuasan anda tentang **hubungan seks** dengan pasangan anda?
Silakan tanda [✓] satu kotak sahaja.

Sangat puas .... ☐
Sederhana puas .... ☐
Puas dan tidak puas adalah hampir sama .... ☐
Sederhana tidak puas .... ☐
Sangat tidak puas .... ☐

15. **Dalam tempoh 4 minggu yang lalu**, bagaimanakah anda menilai **keyakinan** anda bahawa anda boleh mendapat dan mengekalkan ereksi (penegakan zakar)?
Silakan tanda [✓] satu kotak sahaja.

Sangat tinggi .... ☐
Tinggi .... ☐
Sederhana .... ☐
Rendah .... ☐
Sangat rendah. .... ☐

* **Persetubuhan**: Ditakrifkan sebagai penembusan (kemasukan) ke dalam faraj pasangan anda.
** **Aktiviti seks**: Termasuk persetubuhan, bercumbu-cumbuan, permulaan dalam perbuatan seks dan pelancapan.
*** **Pancutan mani**: Ditakrifkan sebagai pemancutan air mani daripada zakar (atau rasa seperti itu).
**** **Rangsangan seks**: Termasuk situasi seperti permainan yang memberahikan bersama pasangan anda, melihat pada gambar-gambar yang memberahikan (contohnya filem atau fotograf) dan sebagainya.
